# Revisiting the antiangiogenic mechanisms of fluorinated thalidomide derivatives

**DOI:** 10.1016/j.bmcl.2024.129858

**Published:** 2024-06-23

**Authors:** Johannes Sievers, Rabea Voget, Feiteng Lu, Kathleen M. Garchitorena, Yuen Lam Dora Ng, Cindy H. Chau, Christian Steinebach, William D. Figg, Jan Krönke, Michael Gütschow

**Affiliations:** a Pharmaceutical Institute, Pharmaceutical and Medicinal Chemistry, University of Bonn, An der Immenburg 4, D-53121 Bonn, Germany; b Department of Hematology, Oncology, and Cancer Immunology, Charité – Universitäatsmedizin Berlin, D-12203 Berlin, Germany; c Molecular Pharmacology Section, Genitourinary Malignancies Branch, Center for Cancer Research, National Cancer Institute, National Institutes of Health, Bethesda, MD, USA

**Keywords:** Angiogenesis, Cancer therapy, Cereblon, Immunomodulatory drugs, Neosubstrates

## Abstract

Introduction of fluorine into bioactive molecules has attracted much attention in drug development. For example, tetrafluorination of the phthalimide moiety of immunomodulatory drugs (IMiDs) has a strong beneficial effect on the ability to inhibit angiogenesis. The neomorphic activity of E3 ligase complexes is induced by the binding of IMiDs to cereblon. We investigated that a set of eight thalidomide analogs, comprising non- and tetrafluorinated counterparts, did not induce the degradation of neomorphic substrates (IKZF3, GSPT1, CK1α, SALL4). Hence, the antiangiogenic activity of fluorinated IMiDs was not triggered by neosubstrate degradation features. A fluorine scanning of non-traditional IMiDs of the benzamido glutarimide chemotype was performed. By measuring the endothelial cell tube formation, no angiogenesis inhibitors were identified, confirming the narrow structure–activity window of IMiD-induced antiangiogenesis.

Angiogenesis, the process of generating new blood vessels from pre-existing capillaries and eventually creating a complete vascular network, is a hallmark of cancer. The tumor microenvironment initiates the complex process of angiogenesis that is stimulated through the activation of vascular permeability factors, such as VEGF, and involves proteolysis, migration, invasion, tubule formation, and cell proliferation. As blood vessels are required to supply oxygen and nutrients for tumor proliferation and expansion, antiangiogenic therapy has been defined as a valuable strategy for cancer treatment.^1^ Immunomodulatory drugs (IMiDs), such as thalidomide or lenalidomide, are widely applied for the therapy of multiple myeloma and other hematologic malignancies. IMiDs exert antiangiogenic effects, as shown for thalidomide already in 1994,^2^ and tailored molecular modifications provided much more potent inhibitors of angiogenesis, in particular, tetrafluorinated analogs.^3–6^

Cereblon (CRBN) forms an E3 ligase complex, referred to as CRL4^CRBN^, together with DDB1, CUL4, and RBX1, and functions as a substrate receptor of this complex. IMiDs act as molecular glue degraders by recruiting target proteins to this E3 ligase. Upon binding to CRBN, IMiDs create a neomorphic interface that is recognized by neosubstrates *via* a structurally conserved β-harpin motif, *i.e.*, an eight-amino-acid stretch with an invariant glycine facing directly the IMiD.^7,8^ Thereby, IMiDs commit *e.g.*, Ikaros (IKZF1), Aiolos (IKZF3), casein kinase 1α (CK1α), G1 to S phase transition protein 1 (GSTP1), or spalt-like transcription factor 4 (SALL4) to ubiquitin-mediated proteasomal degradation.^9,10^ Hence, this class of drugs, in particular high-affinity representatives, are called CRBN E3 ligase modulators (CeLMoDs). Drug-induced neosubstrate degradation has contradictory therapeutic consequences. In multiple myeloma treatment, for example, degradation of the zinc-finger transcription factors IKZF1 and IKZF3 accounts for the anti-myeloma effects of IMiDs, whereas their teratogenic effect is likely to be based on the degradation of SALL4.^10^

Whether neosubstrate profiles of IMiDs affect antiangiogenic activity has not been comprehensively elucidated. At least, SALL4 was reported to be involved in cell proliferation, migration, and tube formation, and to promote angiogenesis by transcriptionally regulating VEGF expression, suggesting its role in tumor vascularization.^11^ However, CRBN was dispensable for microvessel formational in an *ex vivo* mouse aortic ring model.^3^ Nevertheless, based on the consideration that the glutarimide ring of thalidomide-type IMiDs is inserted into the tri-tryptophan pocket of CRBN and that the phthalimide part is exposed on the protein surface, subtle differences in the phthalimide were attributed to confer neosubstrate selectivity to CRBN ligands. Structural modifications revealed that, *e.g.*, 5-hydroxylation of thalidomide enhanced SALL4 degradation and 6-fluorination of lenalidomide induced a more robust degradation of CK1α.^12^

CRBN is responsible for the pleiotropic features of IMiDs, but its function in mediating the antiangiogenic effects of IMiDs remains unclear. While the impact of fluorination on the antiangiogenic profile of IMiDs has been thoroughly documented, a possible involvement of neosubstrate degradation has not yet been examined. We set out to investigate whether the fluorination of IMiDs would affect the recruitment of CRBN neosubstrates for proteasomal degradation. Moreover, we generated a small library of new fluorinated derivatives as non-traditional CRBN binders and determined their antiangiogenic activity.

In a previous study, the correlation of fluorination, CRBN binding, and antiangiogenic activity of IMiDs was investigated.^13^ For this purpose, a set of compounds was designed (Table 1) originating from the structure of thalidomide (**1**) and its tetrafluoro analog (**2**), whose glutarimide was exchanged for a barbituric acid moiety in compounds **3** and **4**, respectively (Scheme S1 in Supplementary Data). One carbonyl group of the five-membered ring was excised in each case, while the substitution pattern was otherwise maintained, leading to the open-chain analogs **5**–**8**. Employing a microscale thermophoresis (MST) assay, the affinity of the compounds to the thalidomide binding domain (TBD) was determined. Exclusively, the glutarimide-containing compounds (**1**, **2**, **5**, **6**) showed binding to TBD (Table 1). The phthalimide derivatives (**1** and **2**) with single-digit micromolar *K*_i_ values outperformed their benzamide counterparts (**5** and **6**). In the endothelial cell tube formation assay, only two compounds (**2** and **4**) out of the set (**1**–**8**) exhibited antiangiogenic activity. The fluorination at the phthalimide core appeared advantageous for the inhibition of angiogenesis, as it has also been shown for tetrafluorophthalimides with *N*-substituents other than glutarimide.^4–6^ Hence, a correlation of the data of tube formation with the results from the MST assay was not assessed, from which it was concluded that the affinity to CRBN appears not to be decisive for the antiangiogenic effects of IMiDs. However, we supposed that fluorination might affect neosubstrate degradation. Therefore, compounds **1**–**8** were subjected to an analysis of their activity to recruit neosubstrate proteins to CRBN for ubiquitination.

Before determining neosubstrate degradation, an obvious effect of fluorination was experimentally specified in this study, *i.e.*, the alteration of lipophilicity. Distribution coefficients at pH 7.4 were determined by an HPLC-based method.^14^ As expected, the lipophilicity (elog*D*_7.4_) increased due to the introduction of fluorine, and elog*D* differences were in the range of 0.8 to 1.2 (Table 1). Phthalimido derivatives **1**–**4** were more lipophilic than the analogous benzamido compounds **5**–**8** whose elog*D* values were lower by 0.5 to 1.0. The exchange of the glutarimide in **1**, **2**, **5**, and **6** by the barbituric acid unit in **3**, **4**, **7**, and **8** increased lipophilicity by elog*D* differences of 1.3 to 1.7. Drug-membrane interactions were assessed by a high-throughput HPLC method on an Immobilized Artificial Membrane (IAM) column consisting of monolayers of phospholipids covalently bound to silica particles.^15^ Chromatographic hydrophobicity index (CHI) values are listed in Table 1.^16^ It is likely that the enhanced membrane affinity of tetrafluorophthalimides **2** and **4**, in comparison with the non-fluorinated analogs **1** and **3**, accounted for the inhibition of tube formation through improved passive diffusion of **2** and **4**.

We also investigated the effect of fluorination on metabolic microsomal stability.^17^ Verapamil, used as a high-clearance positive control to confirm the microsomal CYP enzyme activity, exhibited an *in vitro* halflife of 19.3 min and an intrinsic clearance (Cl_int_) value of 71.7 μL/min/mg protein. Both the antiangiogenic agent **4** and its non-fluorinated counterpart **3** were metabolically stable (*t*_1/2_ > 60 min) and showed a similar clearance (**3**: Cl_int_ = 10.3 μL/min/mg protein; **4:** Cl_int_ = 4.6 μL/min/mg protein).

The antiproliferative activity of compounds **1**–**8** was evaluated in both human umbilical vein endothelial cells (HUVECs) and multiple myeloma MM1.S cells using the CCK-8 cell proliferation assay.^18,19^ Treatment with 100 μM thalidomide (**1**) decreased neither HUVEC proliferation, consistent with previous results,^4^ nor proliferation of MM1.S cells (Fig. S1). While **3** and **5**–**8** did not affect HUVEC or MM1.S cell growth at 10 μM, **2** and **4** showed antiproliferative effects. Tetrafluoro-thalidomide (**2**) reduced HUVEC growth by 48% and MM1.S growth by 95%. Compound **4** exhibited potent antiproliferative activity (>95%) in both HUVEC and MM1.S cells (Fig. S1).

Next, by employing the set of compounds **1**–**8**, we investigated whether fluorination controlled the selectivity of neosubstrate degradation (Fig. 1, Table 1).^20^ MM.1S cells or HuH6 cells were treated with **1**–**8**, as well as the IMiDs lenalidomide, pomalidomide, and mezigdomide (CC-92480). As reported,^14^ the latter three compounds degraded IKZF3 and SALL4. In particular, the mezigdomide-induced degradation of IKZF3 was pronounced, in line with its increased depth, rate, and duration of IKAROS protein degradation, compared to other IMiDs.^21^ In contrast, when MM.1S or HuH6 cells were subjected to treatment with compounds **1**–**8** for a period of 16 h and protein levels were assessed by Western blot, neither the levels of the lymphoid transcription factor IKZF3 nor those of the neosubstrates CK1a, GSPT1 and SALL4 were altered. We also employed a lentiviral reporter vector that ectopically expresses an IKZF3-GFP fusion protein (Figs. S3 and S4).^22^ This method distinguished direct effects on protein degradation from effects that regulate transcriptional expression. MM.1S cells expressing IKZF3-ZF2 constructs in the degradation reporter were treated with **1**–**8** or DMSO and analyzed by flow cytometry to quantify the DMSO-normalized ratio of eGFP/mCherry fluorescence (Fig. S3). The abundance of IKZF3-GFP was not significantly changed by test compounds **2**–**8** which were applied at three different concentrations, *i.e.*, 0.1 μM, 1.0 μM, and 10 μM (Fig. S4). Treatment with thalidomide (**1**) resulted in a concentration-dependent IKZF3 degradation, which was induced to a greater extent by lenalidomide, pomalidomide, and mezigdomide treatment. Noteworthy, the angiogenesis inhibitors **2** and **4** did not modulate neosubstrate degradation. These data strongly suggest that the antiangiogenic effects of IMiDs are not controlled through an alteration of CRBN neosubstrates.

IMiDs are accommodated in a conserved hydrophobic tri-tryptophan pocket in the C-terminal domain of CRBN through H-bonds promoted interactions of their glutarimide moiety (Fig. 2). The endogenous degron motif, a C-terminal cyclic imide, binds to the same tryptophan cage. Proteins are equipped with such moieties through intramolecular cyclizations of glutamine or asparagine residues that come along with spontaneous protein chain breaks, forming potentially malfunctional fragments.^23^ These findings on the natural CRBN degron have laid the foundation for further studies towards the medicinal chemistry-driven optimization of minimal CRBN ligands.^8,14,24,25^.

Whereas the effect of fluorination on the angiogenesis inhibiting properties of prototypical, phthalimide-type IMiDs has thoroughly been studied, data are lacking for modified chemotypes of CRBN binders, *e.g.*, benzamido glutarimides. We have synthetically provided a small series (**9**–**19**) of such benzamides (Table 2), designed to be employed for a fluorine scanning approach (Scheme S1). Compounds **9**–**19** were prepared in 48–74% yield, mainly in a carbodiimide-promoted reaction from the corresponding fluoro-substituted benzoic acids and 3-amino-2,6-piperidinedione hydrochloride.^27,28^ Distribution coefficients and chromatographic hydrophobicity indices were obtained (Table 2) and revealed the expected correlation of these parameters with the number of fluorine atoms incorporated into the aromatic core of benzamido glutarimides (Fig. S5).

The antiangiogenic activity of the compounds was assessed *via* the endothelial cell tube formation assay. HUVECs can form hollow tube-like structures when cultured upon biological gels, such as ECMatrix. The formation of the tubules can then be used as an appropriate *in vitro* measurement of angiogenesis, with the extent of inhibition corresponding to the antiangiogenic effects of the compounds. We have previously optimized the lattice assay for screening a series of phthalimide and benzamide derivatives (**1**–**8**)^13^ and applied this technique for the evaluation of the new benzamides **9**–**19**.^29^ A concentration of 10 μM was used for the initial screening to assess the antiangiogenic activity. All compounds were tested at this dose along with the positive control, *i.e.*, tetrafluoro-thalidomide (**2**) and the previously investigatedbenzamide derivatives **5** and **6** (Figs. S6 and S7). Neither **5** and **6**, in accordance with previous investigations,^13^ nor the new compounds **9**–**19** demonstrated significant antiangiogenic activity at 10 μM in the tube formation assay. All compounds were further tested at 100 μM (Table 2, Figs. S6 and S7), but none of them exhibited antiangiogenic activity greater than 50% inhibition, similar to thalidomide (**1**), which did not demonstrate potent antiangiogenic activity, consistent with previous results showing that **1** failed to significantly block tube formation at a concentration range of 12.5–100 μM.^30^ In fact, the great potency of tetrafluoro-thalidomide (**2**) was confirmed in the course of this study.

The *N*-connected glutarimide moiety constituted the common structural feature of the investigated compounds **1**, **2**, **5**, **6**, and **9**–**19**. However, only when combined with the bicyclic tetrafluorophthalimide core as realized in **2**, antiangiogenic activity was achieved. Otherwise, a glutarimide moiety is not necessarily required for antiangiogenic properties of IMiDs. Several tetrafluorophthalimide derivatives have been reported to inhibit angiogenesis, including those with *N*-alkyl and *N*-aryl substituents,^4,5^ as well as tetrafluorophthalimides with a barbituric acid portion.^6^

We also investigated homo-PROTAC **20** (Table 2), a potent CRBN degrader,^31^ together with the antiangiogenic agent CPS-49.^32^ PROTAC **20** was not active in the lattice assay at 10 μM and exhibited only a weak effect at 100 μM (Figs. S8 and S9). This result translates to CRBN-independent mechanisms underlying the endothelial cell tube formation. Compound **20** was shown to induce the degradation of the neosubstrate IKZF1, albeit only at 100 μM, but not that of CK1α^31^ underpinning these neosubstrates not to be involved in the endothelial tubule formation.

The chemistry of glutarimides has raised substantial interest in the field of targeted protein degradation due to the utilization of the glutarimide moiety as the key element for cereblon recognition.^8,23^ Alternative, non-phthalimide cereblon ligands have recently been discovered that feature the glutarimide portion connected to an aromatic core, either directly in case of phenyl-glutarimides,^24^ *via* an NH bridge in case of anilino-glutarimides,^25^ or *via* a CONH junction in case of benzamide-based cereblon binders.^14^ The latter type of compound was shown in this study not to inhibit endothelial tube formation. We provide further evidence that the antiangiogenic properties of certain, fine-tuned IMiDs are not mediated by cereblon binding of these compounds and the successive degradation of neosubstrates. These findings point to the ubiquitin-independent pathway through which IMiDs exert anti-angiogenic effects. Hence, chemical properties of IMiDs that are associated with angiogenesis inhibition, on the one hand, and targeted protein degradation, on the other hand, can be independently introduced to advance bioactive compounds into drug candidates.

## Supplementary Material

Supplementary

Supplementary data to this article can be found online at https://doi.org/10.1016/j.bmcl.2024.129858.

## Figures and Tables

**Fig. 1.**
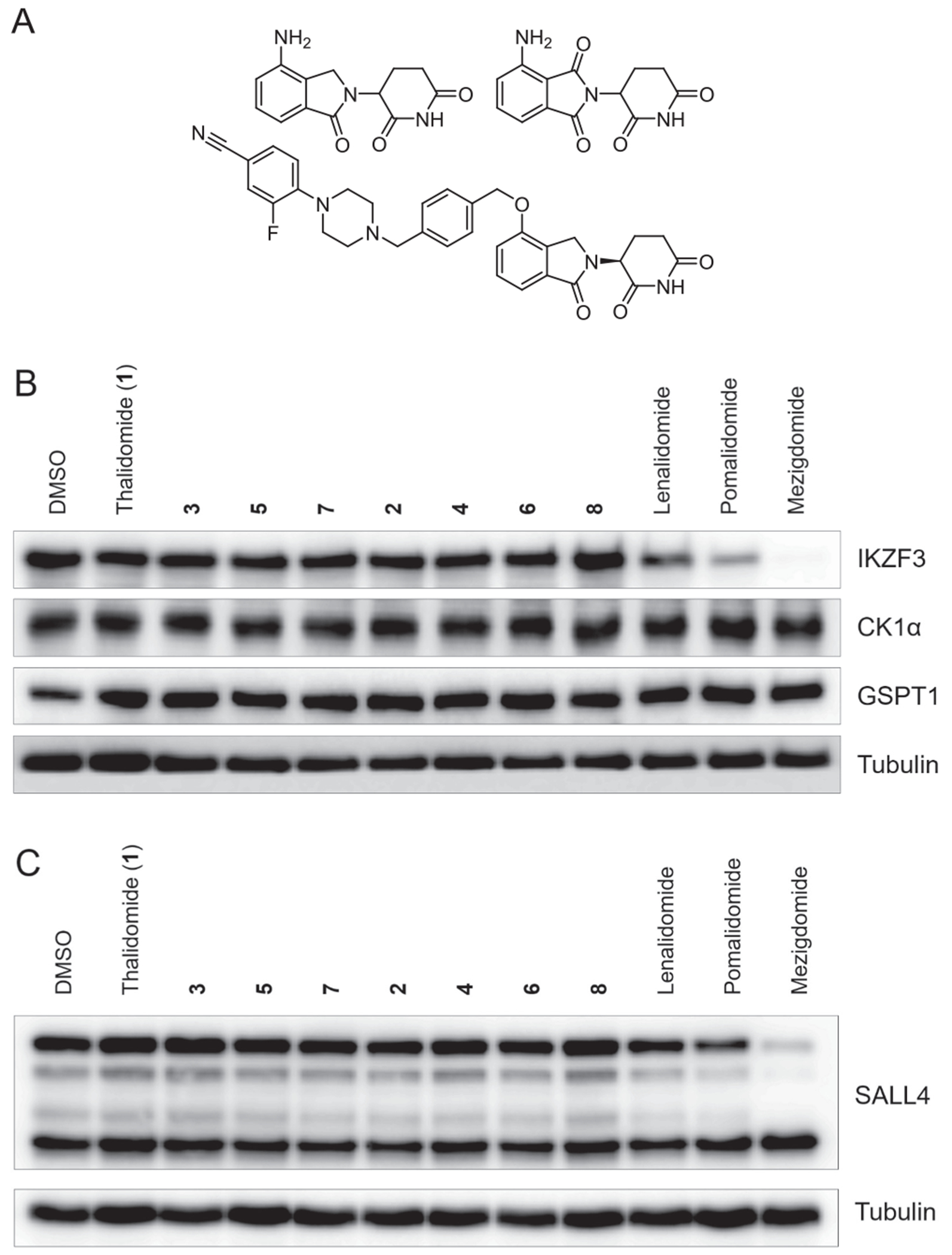
Neosubstrate modulation by compounds **1**–**8** and further IMiDs at a concentration of 1.0 μM each (for Western blots from experiments with a test compound concentration of 0.1 μM each, see Fig, S2). **A**. Chemical structures of lenalidomide (top left), pomalidomide (top right), and mezigdomide (bottom). **B**. MM.1S cells were treated with 1.0 μM of each compound for 16 h before lysis and blotting for IKZF3, GSPT1, and CK1α. **C**. HuH6 cells were treated with 1.0 μM of each compound for 16 h before lysis and blotting for SALL4.

**Fig. 2.**
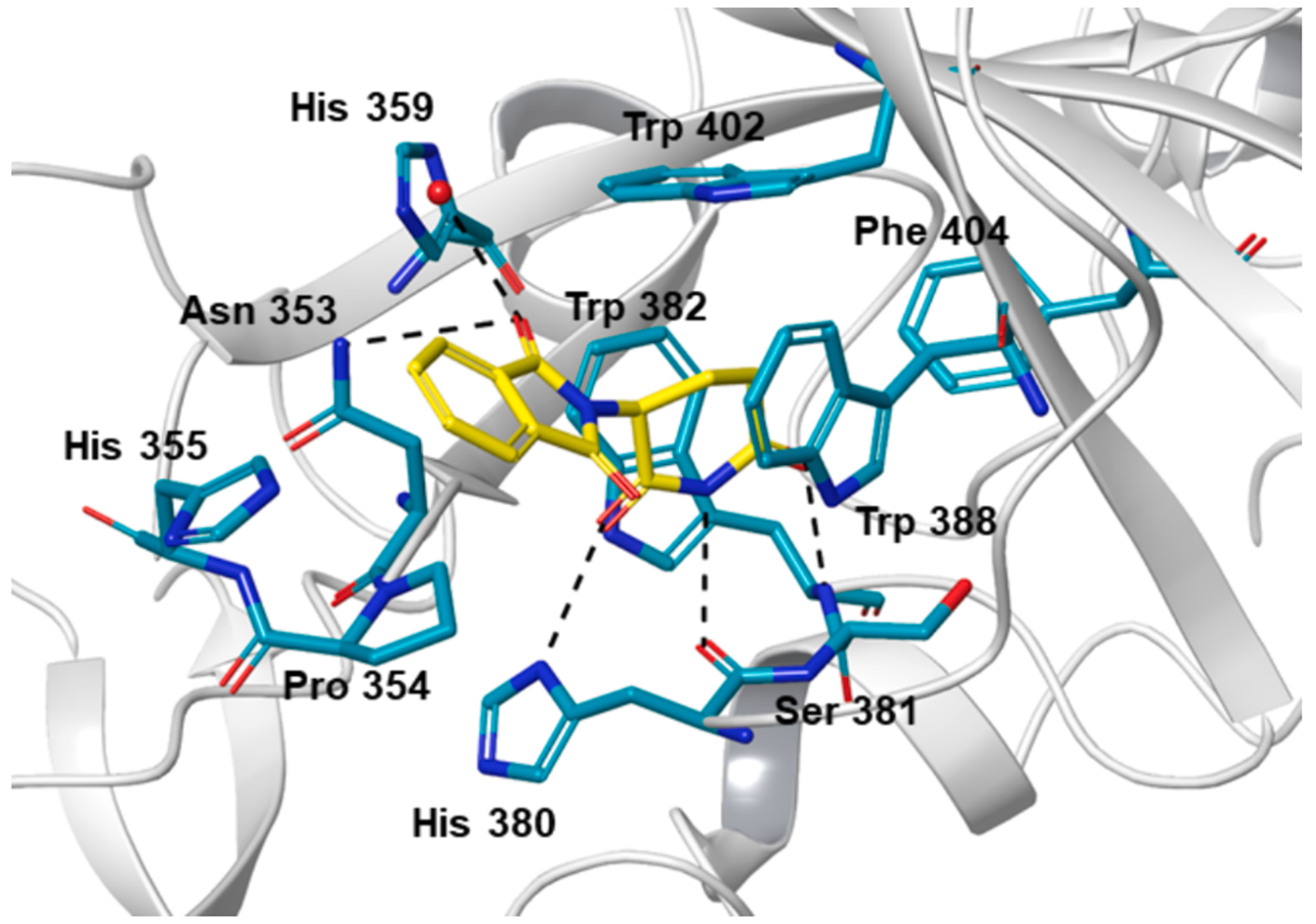
Crystal structure of thalidomide (yellow) in complex with CRBN from *Gallus gallus* (PDB ID 4CI1).^26^ Hydrogen bonds are shown as dashed lines. Thalidomide binds in a highly conserved tri-tryptophan cavity. The glutarimide moiety and one 1*H*-pyrrole-2,5-dione carbonyl group primarily contribute to ligand accommodation. The protruding benzene core of the phthalimide moiety is not involved in binding. The figure was generated with Maestro (Schrödinger). (For interpretation of the references to colour in this figure legend, the reader is referred to the web version of this article.)

**Table 1:** Antiangiogenic and CRBN-binding Activities, Distribution Coefficients, and Hydrophobicity Indices of Phthalimidoglutarimides **1** and **2**, Phthalimidobarbituric Acids **3** and **4**, Benzamidoglutarimides **5** and **6**, Benzamidobarbituric Acids **7** and **8**.

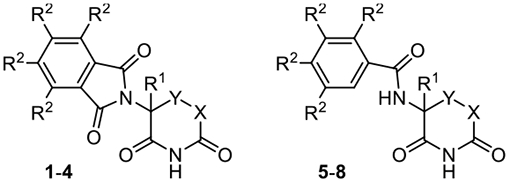										

Cmpd	X	Y	R^1^	R^2^	Tube formation IC_50_ (μM)^a^	MST *K*_i_ (μM)^b^	elog*D*_7.4_^c^	CHI_IAM_^d^	Neosubstrate degradation (%)	
									IKZF3^e,g^	SALL4^f,g^
**1**	–CH_2_–	–CH_2_–	H	H	n.i.^h^	8.55	0.5	12.5	11	13
**2**	–CH_2_–	–CH_2_–	H	F	3.2	6.08	1.4	17.8	<5	<5
**3**	N-CH(CH_3_)_2_	CO	CH_3_	H	n.i.	n.b.^i^	2.2	28.9	11	<5
**4**	N-CH(CH_3_)_2_	CO	CH_3_	F	2.8	n.b.	3.0	31.6	<5	<5
**5**	–CH_2_–	–CH_2_–	H	H	n.i.	62.6	−0.3^j^	6.3^j^	5	<5
**6**	–CH_2_–	–CH_2_–	H	F	n.i.	30.4	0.9^j^	16.7^j^	<5	<5
**7**	N-CH(CH_3_)_2_	CO	CH_3_	H	n.i.	n.b.	1.2	20.9	<5	<5
**8**	N-CH(CH_3_)_2_	CO	CH_3_	F	n.i.	n.b.	2.2	27.9	<5	<5

a Endothelial cell tube formation assay. Data from lit.^13^

b Affinity to the human TBD (residues 319–425 of human CRBN) was determined through an MST assay. Data from lit.^13^

c Distribution coefficients at pH 7.4 were estimated by an HPLC-based method.

d Chromatographic hydrophobicity index values referring to IAM chromatography (CHI_IAM_ values), an estimate for drug-membrane interactions and permeability.

e Percentage of degraded IKZF3 protein after 16h treatment of MM.1S cells with 1.0 μM of each compound.

f Percentage of degraded SALL4 protein after 16h treatment of HuH6 cells with 1.0 μM of each compound.

g Western blots were analyzed by densitometric methods, and values were normalized to the respective loading controls and to DMSO-treated conditions. IKZF3/SALL4 degradation data represent the average of at least three independent biological experiments.

h n.i., no inhibition. Compounds were tested in the concentration range of 10–100 μM and did not show significant antiangiogenic activity.

i n.b., no binding. No significant CRBN binding was determined.

j Data from lit.^14^

**Table 2:** Structures, Distribution Coefficients, and Hydrophobicity Indices of Benzamidoglutarimides **9**–**19** and Homo-PROTAC **20**.

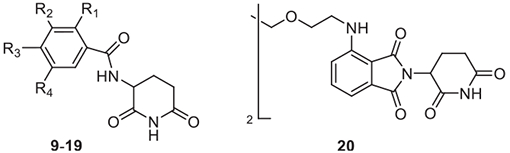							

Compd	R^1^	R^2^	*R* ^3^	R^4^	elog*D*_7.4_^a^	CHI_IAM_^b^	Inhibition of tube formation (%)^c^
**9**	F	H	H	H	−0.1	5.7	13.4
**10**	H	F	H	H	0.1	10.6	10.3
**11**	H	H	F	H	0.1	10.1	17.9
**12**	F	F	H	H	0.3	7.6	31.1
**13**	F	H	F	H	0.3	7.2	35.8
**14**	F	H	H	F	0.3	7.2	24.2
**15**	H	F	H	F	0.6	12.3	44.3
**16**	H	F	F	H	0.6	13.6	15.5
**17**	F	F	F	H	0.7	12.2	30.3
**18**	F	H	F	F	0.6	11.0	n.i.^d^
**19**	H	F	F	F	1.0	17.9	8.1
**20**	–	–	–	–	n.d.^e^	n.d.^e^	50

a Distribution coefficients at pH 7.4 were estimated by an HPLC-based method.

b Chromatographic hydrophobicity index values referring to IAM chromatography (CHI_IAM_ values), an estimate for drug-membrane interactions and permeability.

c Mean area of lattice formation relative to vehicle control. Compounds were used at a concentration of 100 μM. Compound **2**, as a positive control, showed 95.3% inhibition of tube formation at 10 μM. Thalidomide (**1**), as a comparator, showed 27.8% inhibition of tube formation at 100 μM.

d n.i., no inhibition.

e n.d., not determined.
