# Supplementary material for: Revisiting the antiangiogenic mechanisms of fluorinated thalidomide derivatives
Source: Bioorg Med Chem Lett. Author manuscript; Available in PMC 2025 Jul 24. (PMC12288609; doi:10.1016/j.bmcl.2024.129858)
Supplement: Supplementary [file NIHMS2097134-supplement-Supplementary.pdf]

Supplementary Data

Revisiting the antiangiogenic mechanisms of fluorinated thalidomide derivatives

Johannes Sievers, Rabea Voget, Feiteng Lu, Kathleen M. Garchitorena, Yuen Lam Dora Ng, Cindy H. Chau, Christian Steinebach, William D. Figg, Jan Krönke, and Michael Gütschow*

Table of Content

1. **Supplementary Figures and Schemes**………………………………………………….……... S2
   - **Figure S1.** Antiproliferative effects of compounds **1-8**……………………….……….. S2
   - **Figure S2.** Neosubstrate modulation by **1-8** and further IMiDs at 0.1 µM……………….. S3
   - **Figure S3.** Flow-cytometry-based analysis of IKZF3-ZF2 degradation.………………..…… S4
   - **Figure S4.** Quantification of the IKZF3 degradation……………………………………… S5
   - **Figure S5.** Correlation of the number of F atoms with elog$D_{7.4}$ and CHI_{IAM} for **5**, **6**, **9-19**.. S6
   - **Figure S6.** Results of the endothelial tube formation assay with **1**, **2**, **5-19**………………..S7
   - **Figure S7.** Representative images of the tube formation assay with **1**, **2**, **5-19**………...…. S8
   - **Figure S8.** Results of the endothelial tube formation assay with CPS-49, **1**, and **20**……… S9
   - **Figure S9.** Representative images of the tube formation assay with CPS-49, **1**, and **20**….. S9
   - **Scheme S1.** Synthesis of thalidomide derivatives ………………………………………… S10
   - **Table S1.** Analytical data of compounds **9-19**…………………………………………..…. S11
2. **NMR Spectra**…………………………………………………………..…….….…….. S15
3. **References**…………………………………………………………………………...… S26

1. Supplementary Figures

A

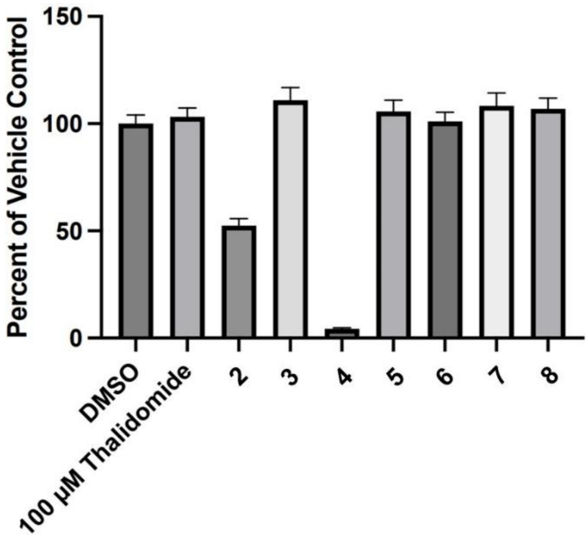

B

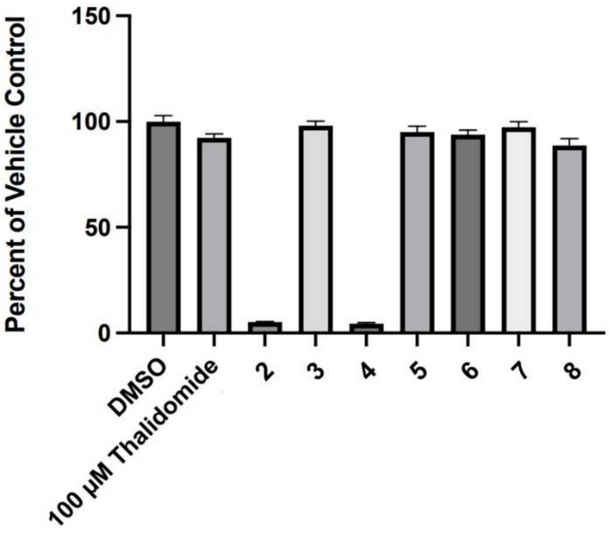

Figure S1. Antiproliferative effects of compounds **1-8** in two cell lines determined by the CCK-8 cell proliferation assay. Cells were treated with 10 µM of the test compound or 100 µM thalidomide (**1**). Data are from at least three replicates from at least three experiments. Vehicle control was 0.5% DMSO. **A.** Human umbilical vein endothelial cells (HUVECs) were treated with the test compounds for 48 hours. **B.** Multiple myeloma MM1.S cells were treated with the test compounds for 72 hours.

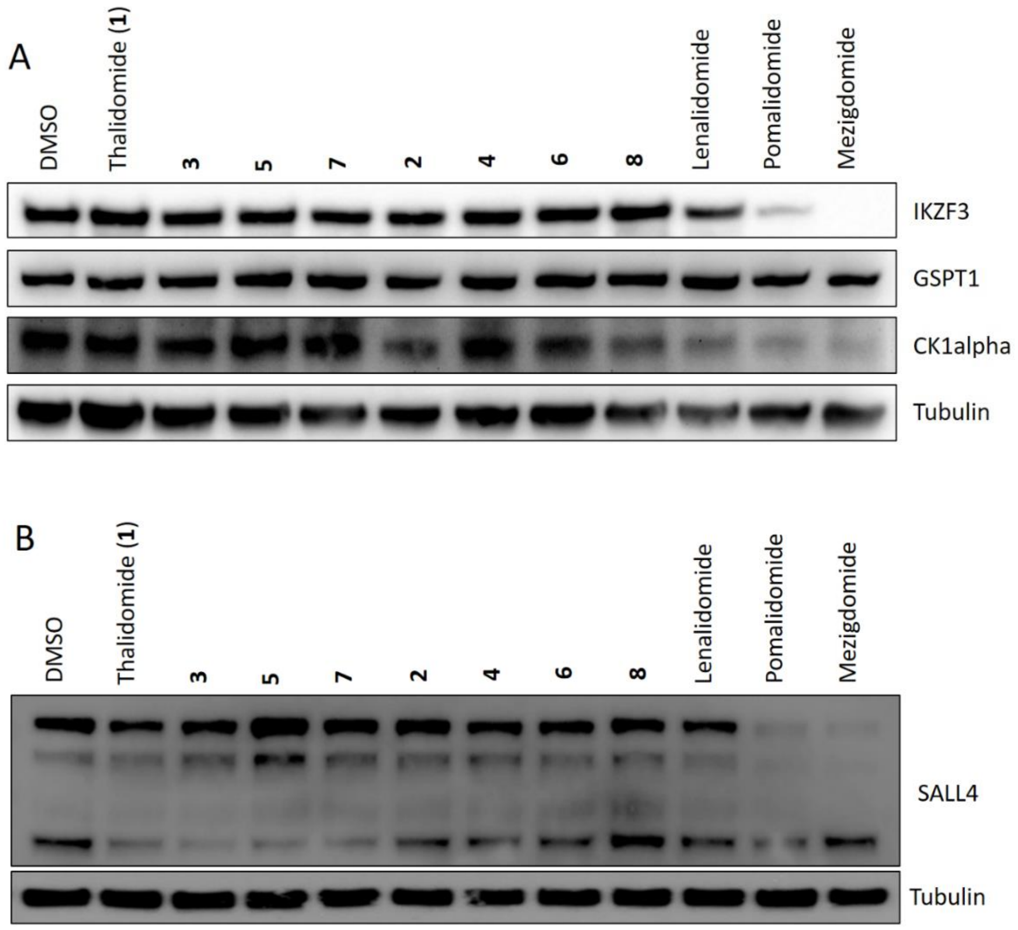

Figure S2. Neosubstrate modulation by compounds **1-8** and further IMiDs. **A**. MM.1S cells were treated with 0.1 μM of each compound for 16 h before lysis and blotting for IKZF3, GSPT1, and CK1α. **B**. HuH6 cells were treated with 0.1 μM of each compound for 16 h before lysis and blotting for SALL4.

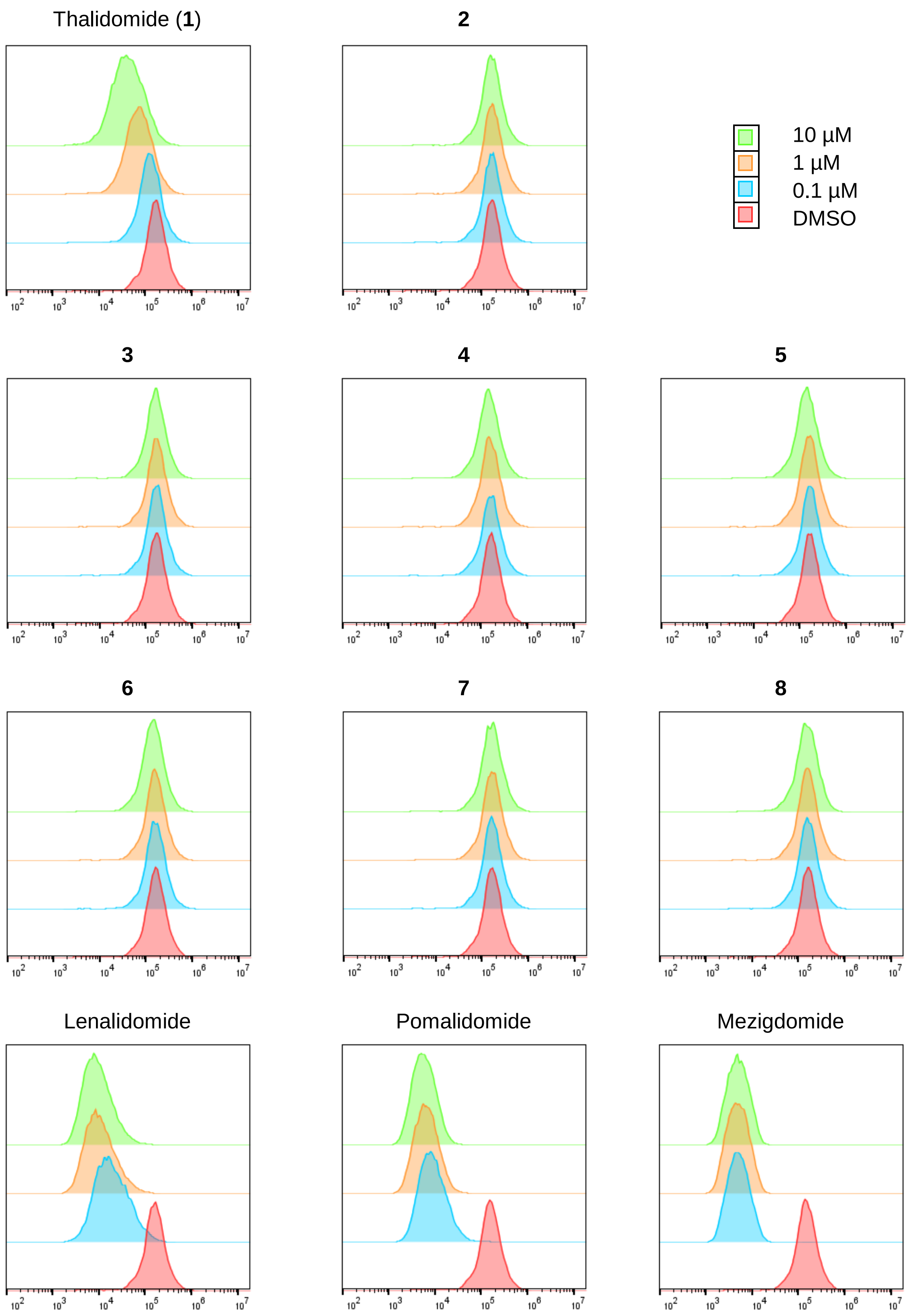

Figure S3. Flow-cytometry-based analysis of IKZF3-ZF2 degradation. MM.1S cells were treated for 16 h with each compound at a concentration of 10, 1.0 and 0.1 μM. The X-axis is indicative of GFP signal from IKZF3-ZF2.

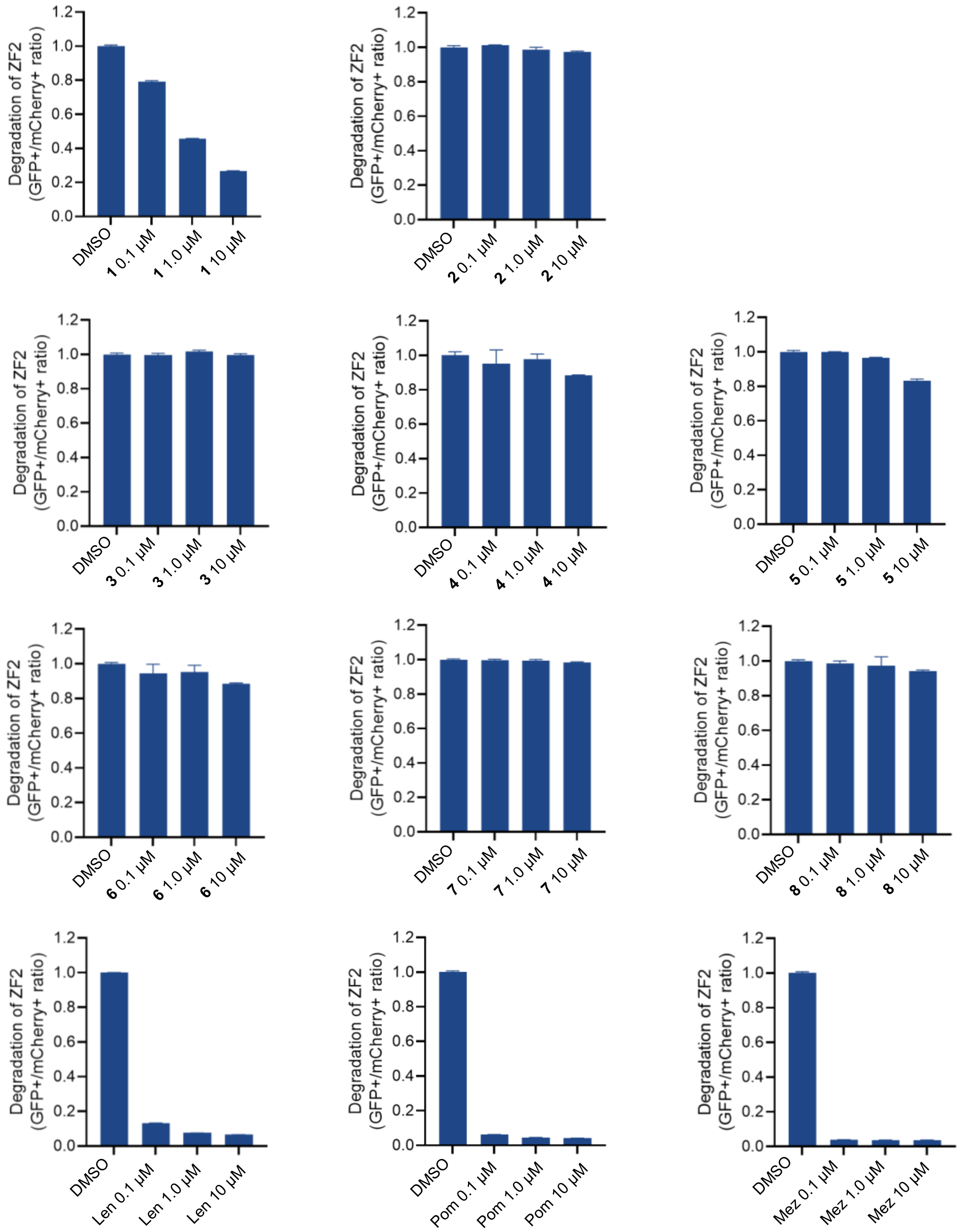

Figure S4. Quantification of the IKZF3 degradation. Data from the ZF2 Artichoke system shown in Figure S2 were used for the quantification of the IKZF3 degradation.

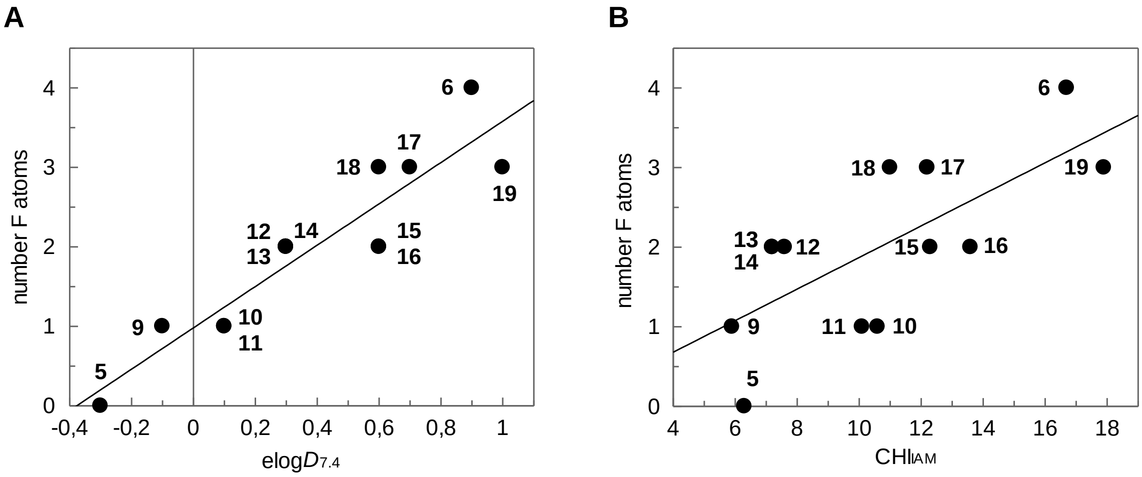

Figure S5. Correlation of the number of fluorine (F) atoms with (**A**) elog$D_{7.4}$ and (**B**) CHI$_{IAM}$ values for **5**, **6**, **9-19**. Linear regression gave R^2 = 0.854 (**A**) and R^2 = 0.500 (**B**).

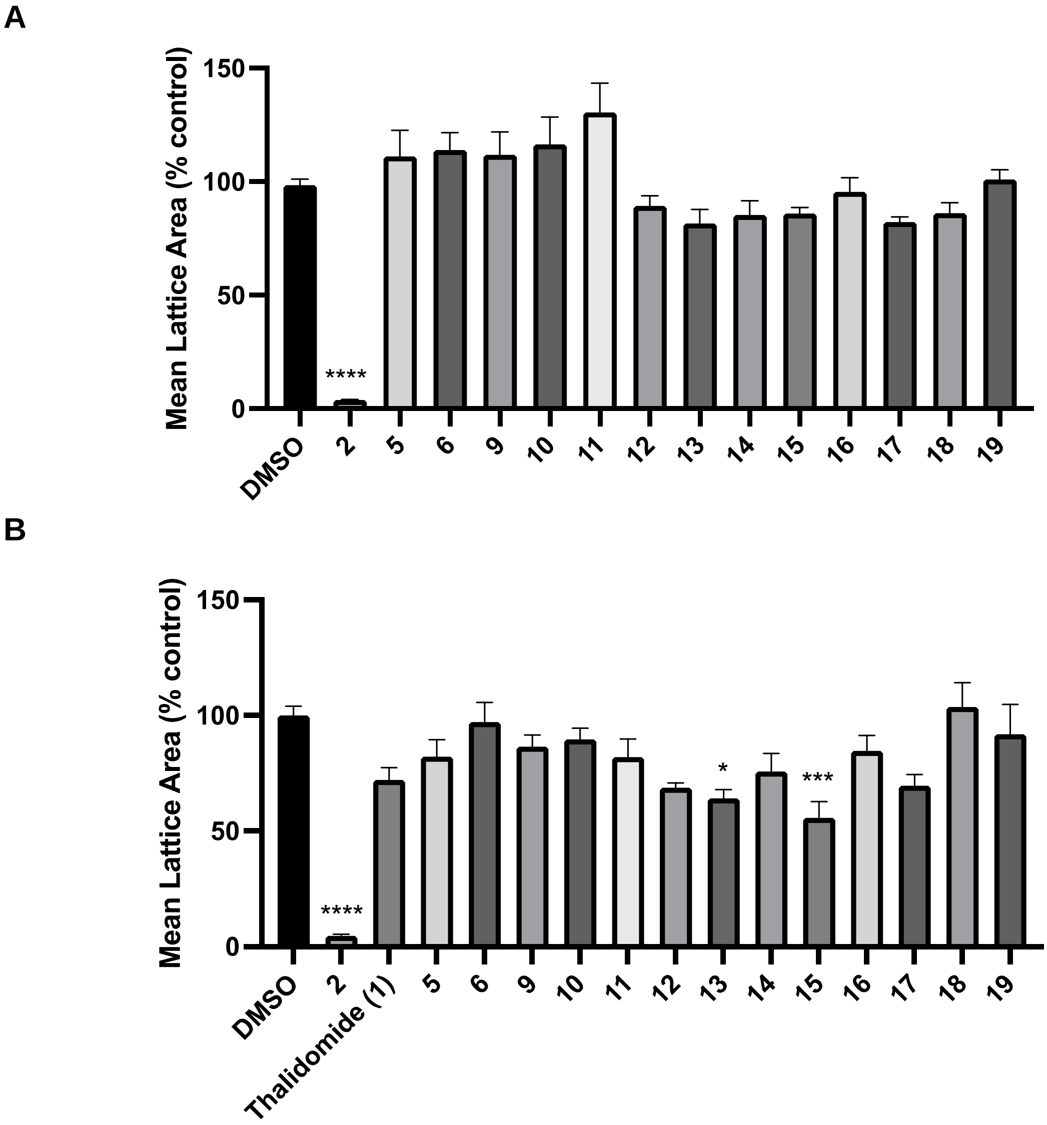

Figure S6. Results of the endothelial tube formation angiogenesis assay in HUVECs upon treatment with test compounds for 18 hours. Vehicle control was 0.1% DMSO. Graphs represent the mean area of lattice formation relative to vehicle control. Statistical significance was assessed using two-tailed Students t-tests and error bars represent mean ± standard error of the mean. **A.** Compounds **2** and **5-19** were tested at 10 µM. Tetrafluoro-thalidomide (**2**) significantly inhibited tubule formation (**** $p < 0.0001$). **B.** Thalidomide (**1**) and compounds **2** and **5-19** were tested at 100 µM. Tetrafluoro-thalidomide (**2**) significantly inhibited tubule formation (**** $p < 0.0001$, *** $p <0.001$, * $p < 0.05$).

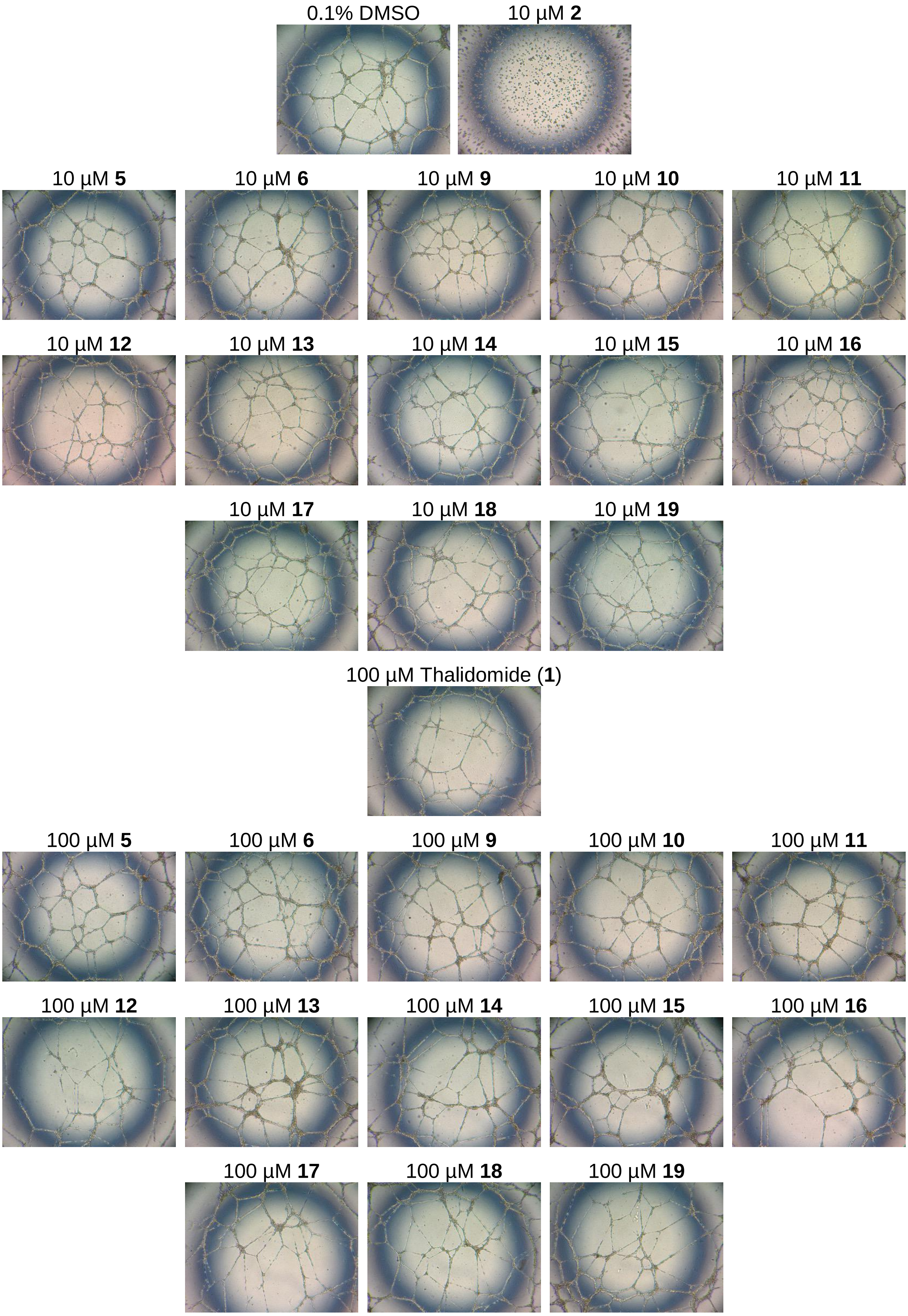

Figure S7. Representative images of the tube formation assay upon treatment with thalidomide (**1**) and compounds **2** and **5-19**.

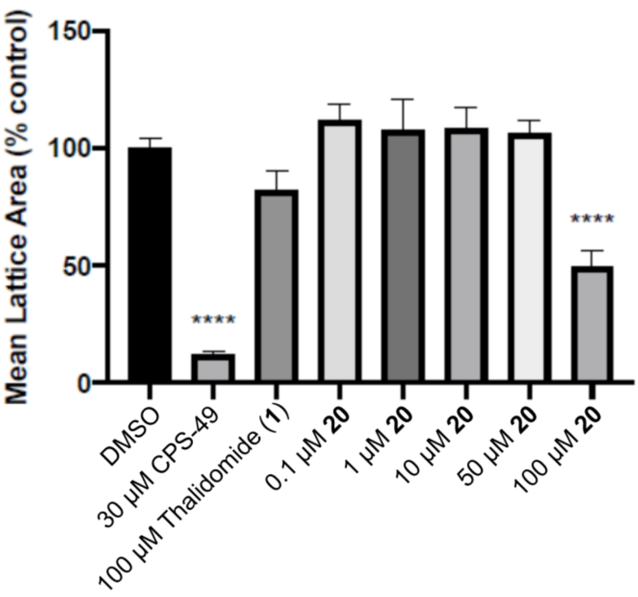

Figure S8. Results of the endothelial tube formation angiogenesis assay in HUVECs upon treatment with CPS-49, thalidomide (**1**), and homo-PROTAC **20**. Vehicle control was 0.5% DMSO. Graphs represent the mean area of lattice formation relative to vehicle control. Statistical significance was assessed using two-tailed Students t-tests and error bars represent mean ± standard error of the mean (**** $p < 0.0001$).

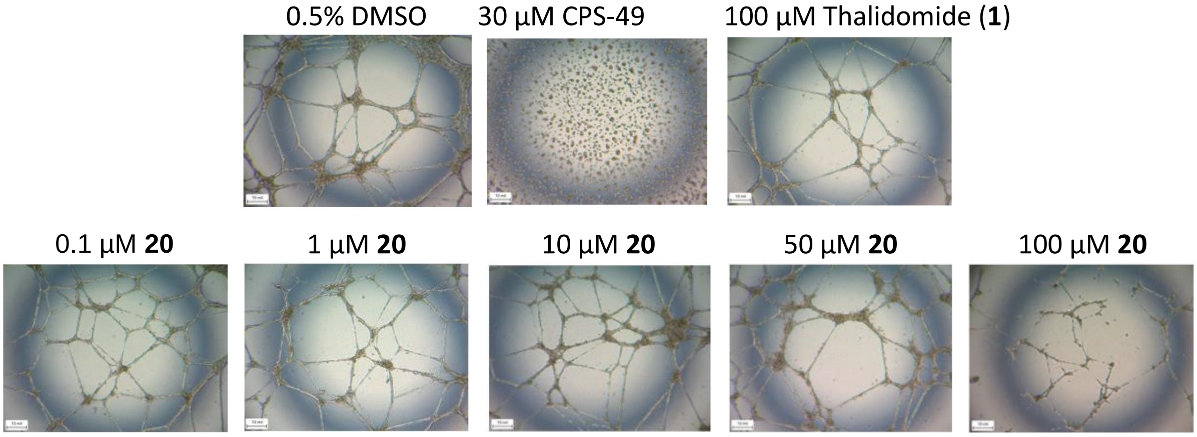

Figure S9. Representative images of the tube formation assay upon treatment with CPS-49, thalidomide (**1**), and PROTAC **20**.

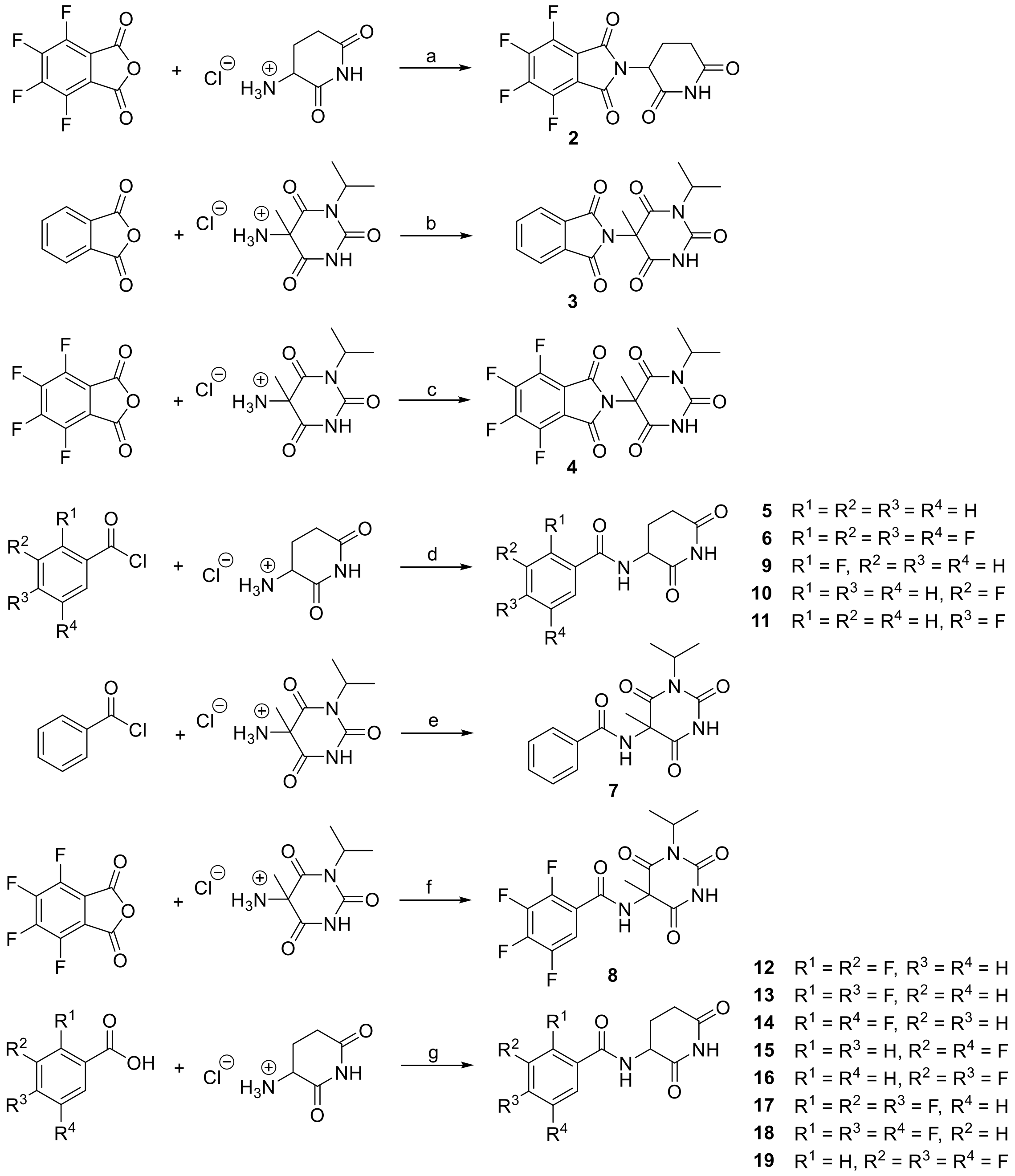

Scheme S1. Synthesis of thalidomide derivatives. Thalidomide (**1**) was purchased from Tocris Bioscience (U.K.). Reagents and conditions a) AcOH, NaOAc, 118 °C, 3 h (Heim *et al*., 2021; Burslem *et al*. 2018); b) Et_3N, DMF, 153 °C, 5 h (Ambrożak *et al.* 2016); c) Et_3N, AcOH, 118 °C, 3 h (Ambrożak *et al*. 2016); d) Et_3N, CH_2Cl_2, 0 °C to rt, 18 h (Heim *et al*., 2021; Steinebach *et al.* 2023); e) Et_3N, CH_2Cl_2, 0 °C to rt, 18 h (Heim *et al*., 2021); f) Et_3N, DMSO, 153 °C, 5 h (Ambrożak *et al*. 2016); g) *N*,*N*-diisopropylethylamine, 1-ethyl-3-(3-dimethylaminopropyl)carbodiimide hydrochloride, 1*H*-1,2,3-benzotriazol-1-ol monohydrate, DMF, rt, 18 h.

Table S1. Analytical data of compounds **9-19**

Compound	Yield / Mp	NMR data	LC-MS / HRMS data
N-(2,6-Dioxo-3-piperidyl)-2-fluorobenzamide (**9**)	Yield 68%, Mp 148-150 °C	^{1}H NMR (500 MHz, DMSO-d_6) δ 1.96-2.06 (m, 1H), 2.04-2.16 (m, 1H), 2.50-2.57 (m, 1H), 2.72-2.83 (m, 1H), 4.71-4.80 (m, 1H), 7.26-7.33 (m, 2H), 7.51-7.59 (m, 1H), 7.63-7.70 (m, 1H), 8.53-8.55 (m, 1H), 10.83 (s, 1H), ^{13}C NMR (126 MHz, DMSO-d_6) δ 24.16, 31.02, 49.80, 116.35 (d, ^{2}J (C,F) = 22.3 Hz), 123.39 (d, ^{2}J (C,F) = 14.0 Hz), 124.66 (d, ^{3}J (C,F) = 3.4 Hz), 130.38 (d, ^{4}J (C,F) = 2.8 Hz), 132.95 (d, ^{3}J (C,F) = 8.5 Hz), 159.41 (d, ^{1}J (C,F) = 249.6 Hz), 163.68, 171.93, 173.04	LC-MS (ESI) 99% purity, *m/z* $[M+H]^+$ 251.0
N-(2,6-Dioxo-3-piperidyl)-3-fluorobenzamide (**10**)	Yield 64%, Mp 218-220 °C	^{1}H NMR (500 MHz, DMSO-d_6) δ 1.94-2.03 (m, 1H), 2.06-2.18 (m, 1H), 2.50-2.59 (m, 1H), 2.74-2.85 (m, 1H), 4.73-4.82 (m, 1H), 7.37-7.44 (m, 1H), 7.51-7.59 (m, 1H), 7.62-7.69 (m, 1H), 7.73 (dm, ^{3}J = 7.7 Hz, 1H), 8.84 (d, ^{3}J = 8.3 Hz, 1H), 10.84 (s, 1H), ^{13}C NMR (126 MHz, DMSO-d_6) δ 24.24, 31.09, 49.78, 114.23 (d, ^{2}J (C,F) = 22.9 Hz), 118.54 (d, ^{2}J (C,F) = 21.0 Hz), 123.61 (d, ^{4}J (C,F) = 2.8 Hz), 130.73 (d, ^{3}J (C,F) = 8.0 Hz), 136.42 (d, ^{3}J (C,F) = 6.8 Hz), 162.13 (d, ^{1}J (C,F) = 244.3 Hz), 164.96 (d, ^{4}J (C,F) = 2.6 Hz), 172.13, 173.10	LC-MS (ESI) 99% purity, *m/z* $[M+H]^+$ 251.1
N-(2,6-Dioxo-3-piperidyl)-4-fluorobenzamide (**11**)	Yield 57%, Mp 248-252 °C	^{1}H NMR (600 MHz, DMSO-d_6) δ 1.94-2.01 (m, 1H), 2.06-2.16 (m, 1H), 2.50-2.58 (m, 1H), 2.74-2.83 (m, 1H), 4.73-4.80 (m, 1H), 7.28-7.35 (m, 2H), 7.90-7.97 (m, 2H), 8.78 (d, ^{3}J = 8.3 Hz, 1H), 10.84 (s, 1H) ^{13}C NMR (151 MHz, DMSO-d_6) δ 24.33, 31.14, 49.74, 115.46 (d, ^{2}J (C,F) = 21.9 Hz), 130.14 (d, ^{3}J (C,F) = 9.6 Hz), 130.55 (d, ^{4}J (C,F) = 2.9 Hz), 164.17 (d, ^{1}J (C,F) = 248.7 Hz), 165.23, 172.31, 173.16	LC-MS (ESI) 99% purity, *m/z* $[M+H]^+$ 251.0

N-(2,6-Dioxo-3-piperidyl)-2,3-difluorobenzamide (**12**)	Yield 56%, Mp 192-195 °C	^{1}H NMR (500 MHz, DMSO-d_6) δ 2.00-2.12 (m, 2H), 2.54-2.56 (m, 1H), 2.75-2.82 (m, 1H), 4.74-4.79 (m, 1H), 7.29-7.33 (m, 1H), 7.42-7.45 (m, 1H), 7.55-7.61 (m, 1H), 8.75 (d, ^{3}J = 8.1 Hz, 1H), 10.86 (s, 1H),<br>^{13}C NMR (126 MHz, DMSO-d_6) δ 24.08, 30.97, 49.81, 119.64 (d, ^{2}J (C,F) = 17.2 Hz), 125.09 (d, ^{3}J (C,F) = 6.7 Hz), 125.15, 125.92 (d, ^{2}J (C,F) = 10.9 Hz), 147.36 (dd, ^{1}J (C,F) = 251.7, ^{2}J (C,F) = 13.6 Hz), 149.90 (dd, ^{1}J (C,F) = 246.6, ^{2}J (C,F) = 12.8 Hz), 162.72, 171.69, 172.95	LC-MS (ESI) 100% purity, *m/z* [M+H]$^{+}$ 269.0,<br>HRMS (ESI) *m/z* [M+H]$^{+}$ calcd for $C_{12}H_{10}F_2N_2O_3$: 269.0732, found: 269.0731
N-(2,6-Dioxo-3-piperidyl)-2,4-difluorobenzamide (**13**)	Yield 69%, Mp 192-195 °C	^{1}H NMR (600 MHz, DMSO-d_6) δ 1.98-2.13 (m, 2H), 2.52-2.55 (m, 1H), 2.74-2.81 (m, 1H), 4.72-4.77 (m, 1H), 7.18-7.21 (td, ^{3}J (H,F) = 8.5 Hz, ^{4}J (H,H) = 2.4 Hz, 1H), 7.34-7.38 (m, 1H), 7.72-7.76, (m, 1H), 8.56-8.58 (m, 1H), 10.84 (s, 1H),<br>^{13}C NMR (151 MHz, DMSO-d_6) δ 24.13, 31.04, 49.84, 104.84 (t, ^{2}J = 25.8 Hz), 111.21-113.41 (m), 118.50-124.14 (m), 131.55-132.60 (m), 160.02 (dd, ^{1}J (C,F) = 252.9 Hz, ^{3}J (C,F) = 12.8 Hz), 162.87, 163.72 (dd, ^{1}J (C,F) = 249.2 Hz, ^{3}J (C,F) = 12.1 Hz), 171.92, 173.06	LC-MS (ESI) 100% purity, m/z [M+H]$^{+}$ 269.0,<br>HRMS (ESI) *m/z* [M+H]$^{+}$ calcd for $C_{12}H_{10}F_2N_2O_3$: 269.0732, found: 269.0732
N-(2,6-Dioxo-3-piperidyl)-2,5-difluorobenzamide (**14**)	Yield 57%, Mp 196-199 °C	^{1}H NMR (600 MHz, DMSO-d_6) δ 1.99-2.12 (m, 2H), 2.51-2.55 (m, 1H), 2.75-2.81 (m 1H), 4.73-4.78 (m, 1H), 7.36-7.45 (m, 3H), 8.65-8.67 (m, 1H), 10.85 (s, 1H),<br>^{13}C NMR (151 MHz, DMSO-d_6) δ 24.09, 31.01, 49.88, 116.45 (dd, ^{2}J (C,F) = 25.4 Hz, ^{3}J (C,F) = 3.2 Hz), 118.36 (dd, ^{2}J (C,F) = 25.7 Hz, ^{3}J (C,F) = 8.3 Hz), 119.51 (dd, ^{2}J (C,F) = 24.1 Hz, ^{3}J (C,F) = 9.1 Hz), 124.76 (dd, ^{2}J (C,F) = 16.9 Hz, ^{3}J (C,F) = 7.1 Hz), 155.57 (dd, ^{1}J (C,F) = 247.9 Hz, ^{4}J (C,F) = 1.5 Hz), 157.90 (dd, ^{1}J (C,F) = 242.4 Hz, ^{4}J (C,F) = 1.5 Hz), 162.52, 171.81, 173.04	LC-MS (ESI) 100% purity, *m/z* [M+H]$^{+}$ 269.0,<br>HRMS (ESI) *m/z* [M+H]$^{+}$ calcd for $C_{12}H_{10}F_2N_2O_3$: 269.0732, found: 269.0730

N-(2,6-Dioxo-3-piperidyl)-3,5-difluorobenzamide (**15**)	Yield 67%, Mp 201-203 °C	^{1}H NMR (600 MHz, DMSO-d_6) δ 1.96-2.14 (m, 2H), 2.53-2.57 (m, 1H), 2.76-2.82 (m, 1H), 4.75-4.80 (m, 1H), 7.47-7.50 (tt, ^{3}J (H,F) = 9.1 Hz, ^{4}J (H,H) = 2.4 Hz, 1H), 7.55-7.61 (m, 2H), 8.93 (d, ^{3}J = 8.2 Hz, 1H), 10.87 (s, 1H),<br>^{13}C NMR (151 MHz, DMSO-d_6) δ 24.16, 31.07, 49.89, 107.18 (t, ^{2}J (C,F) = 25.9 Hz), 110.86 (dd, ^{2}J (C,F) = 21.1 Hz, ^{4}J (C,F) = 6.0 Hz), 137.53 (t, ^{3}J (C,F) = 8.5 Hz), 162.43 (dd, ^{1}J (C,F) = 247.4 Hz, ^{3}J = 12.9 Hz), 163.76, 171.97, 173.09	LC-MS (ESI) 99% purity, *m/z* $[M+H]^+$ 269.1,<br>HRMS (ESI) *m/z* $[M+H]^+$ calcd for $C_{12}H_{10}F_2N_2O_3$: 269.0732, found: 269.0730
N-(2,6-Dioxo-3-piperidyl)-3,4-difluorobenzamide (**16**)	Yield 74%, Mp 208-210 °C	^{1}H NMR (500 MHz, DMSO-d_6) δ 1.99-2.12 (m, 2H), 2.51-2.83 (m, 2H), 4.74-4.79 (m, 1H), 7.55-7.60 (m, 1H), 7.75-7.78 (m, 1H), 7.88-7.94 (m, 1H), 8.88 (d, ^{3}J = 8.3 Hz, 1H), 10.86 (s, 1H),<br>^{13}C NMR (126 MHz, DMSO-d_6) δ 24.20, 31.05, 49.83, 116.83 (d, ^{2}J (C,F) = 18.4 Hz), 117.76 (d, ^{2}J (C,F) = 17.6 Hz), 124.91 (dd, ^{3}J = 7.3 Hz, ^{4}J (C,F) = 3.3 Hz), 131.45, 149.29 (dd, ^{1}J (C,F) = 246.8 Hz, ^{2}J (C,F) = 12.9 Hz), 151.59 (dd, ^{1}J (C,F) = 250.7 Hz, ^{2}J (C,F) =12.5 Hz), 164.09, 172.02, 173.02	LC-MS (ESI) 100% purity, *m/z* $[M+H]^+$ 269.1
N-(2,6-Dioxo-3-piperidyl)-2,3,4-trifluorobenzamide (**17**)	Yield 60%, Mp 203-205 °C	^{1}H NMR (600 MHz, DMSO-d_6) δ 1.99-2.12 (m, 2H), 2.51-2.55 (m, 1H), 2.75-2.81 (m, 1H), 4.73-4.78 (m, 1H), 7.40-7.45 (m, 1H), 7.49-7.53 (m, 1H), 8.74-8.75 (d, ^{3}J = 8.1 Hz, 1H), 10.86 (s, 1H),<br>^{13}C NMR (151 MHz, DMSO-d_6) δ 24.08, 31.02, 49.89, 113.07 (dd, ^{2}J (C,F) = 17.6 Hz, ^{3}J (C,F) = 3.6 Hz), 121.61, 124.79, 139.23 (dt, ^{1}J (C,F) = 250.2 Hz, ^{2}J (C,F) = 15.7 Hz), 148.63 (ddd, ^{1}J (C,F) = 253.6 Hz, ^{2}J (C,F) = 10.5 Hz, ^{3}J (C,F) = 3.1 Hz), 151.72 (ddd, ^{1}J (C,F) = 250.7 Hz, ^{2}J (C,F) = 9.5 Hz, ^{3}J (C,F) = 2.0 Hz), 162.07, 171.76, 173.03	LC-MS (ESI) 99% purity, *m/z* $[M+H]^+$ 287.0,<br>HRMS (ESI) *m/z* $[M+H]^+$ calcd for $C_{12}H_9F_3N_2O_3$: 287.0638, found: 287.0640

N-(2,6-Dioxo-3-piperidyl)-2,4,5-trifluorobenzamide (**18**)	Yield 54%, Mp 203-206 °C	^{1}H NMR (500 MHz, DMSO-d_6) δ 1.99-2.12 (m, 2H), 2.50-2.55 (m, 1H), 2.74-2.81 (m, 1H), 4.72-4.77 (m, 1H), 7.67-7.75 (m, 2H), 8.65-8.67 (m, 1H), 10.87 (s, 1H), ^{13}C NMR (126 MHz, DMSO-d_6) δ 24.03, 30.95, 49.94, 107.18 (dd, ^{2}J (C,F) = 29.5 Hz, ^{2}J (C,F) = 21.5 Hz), 118.30 (dd, ^{2}J (C,F) = 20.4 Hz, ^{3}J (C,F) = 4.3 Hz), 119.98, 145.89 (ddd, ^{1}J (C,F) = 243.8 Hz, ^{2}J (C,F) = 13.2 Hz, ^{4}J (C,F) = 3.4 Hz), 150.81 (d, ^{1}J (C,F) = 252.0 Hz), 155.13 (d, ^{1}J (C,F) 250.1 Hz), 161.72, 171.69, 172.94	LC-MS (ESI) 100% purity, *m/z* $[M+H]^+$ 287.1, HRMS (ESI) *m/z* $[M+H]^+$ calcd for $C_{12}H_9F_3N_2O_3$: 287.0639, found: 287.0638
N-(2,6-Dioxo-3-piperidyl)-3,4,5-trifluorobenzamide (**19**)	Yield 48%, Mp 208-210 °C	^{1}H NMR (500 MHz, DMSO-d_6) δ 1.96-2.01 (m, 1H), 2.00-2.14 (m, 1H), 2.41-2.57 (m, 1H), 2.76-2.83 (m, 1H), 4.74-4.80 (m, 1H), 7.78-7.84 (m, 2H), 8.94 (d, ^{3}J = 8.3 Hz), 10.88 (s, 1H), ^{13}C NMR (126 MHz, DMSO-d_6) δ 24.12, 31.02, 49.96, 112.51 (dd, ^{2}J (C,F) = 17.3 Hz, ^{3}J (C,F) = 5.1 Hz), 130.32, 141.05 (dt, ^{1}J (C,F) = 254.5 Hz, ^{2}J (C,F) = 15.7 Hz), 150.23 (ddd, ^{1}J (C,F) = 249.1 Hz, ^{2}J (C,F) = 10.1 Hz, ^{3}J (C,F) = 3.4 Hz), 163.08, 171.84, 172.99	LC-MS (ESI) 100% purity, *m/z* $[M+H]^+$ 287.1, HRMS (ESI) *m/z* $[M+H]^+$ calcd for $C_{12}H_9F_3N_2O_3$: 287.0638, found: 287.0641

2. NMR Spectra

^{1}H and ^{13}C NMR spectra of compound **9**

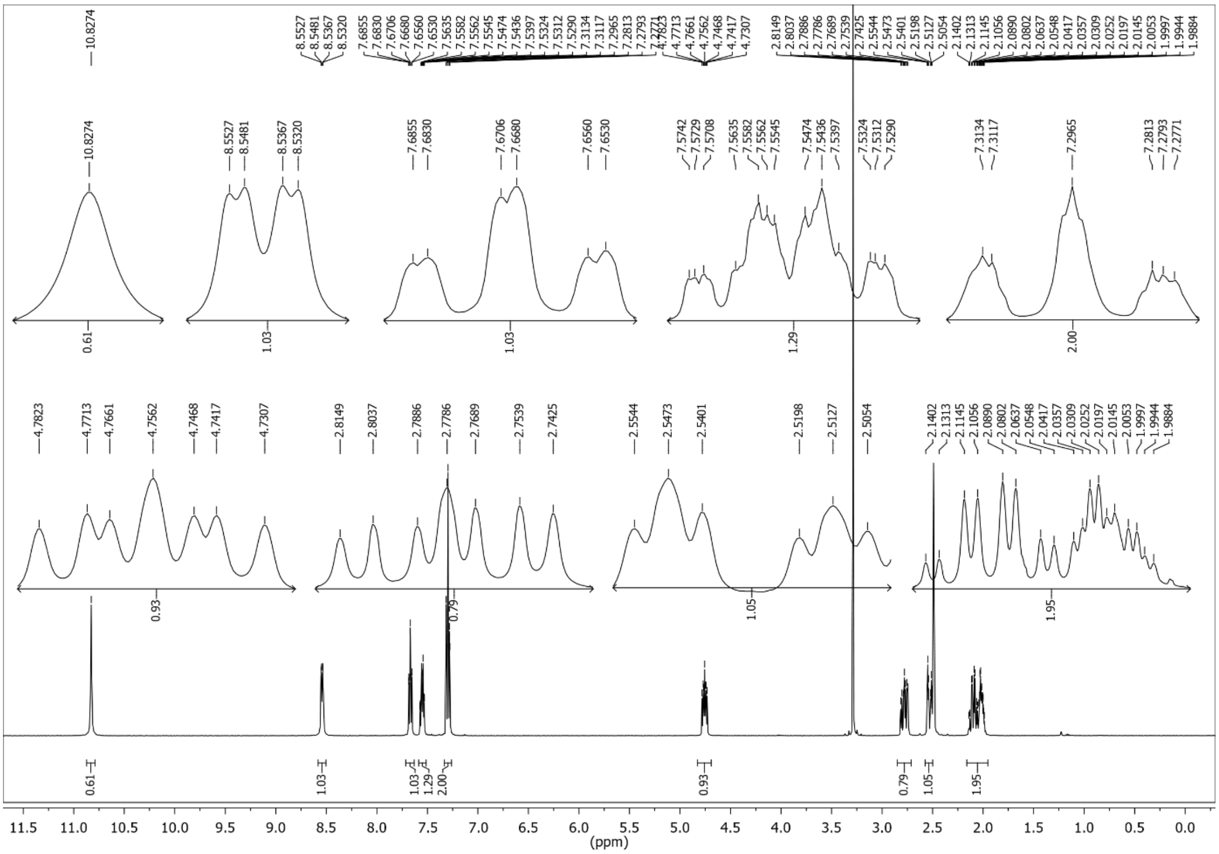

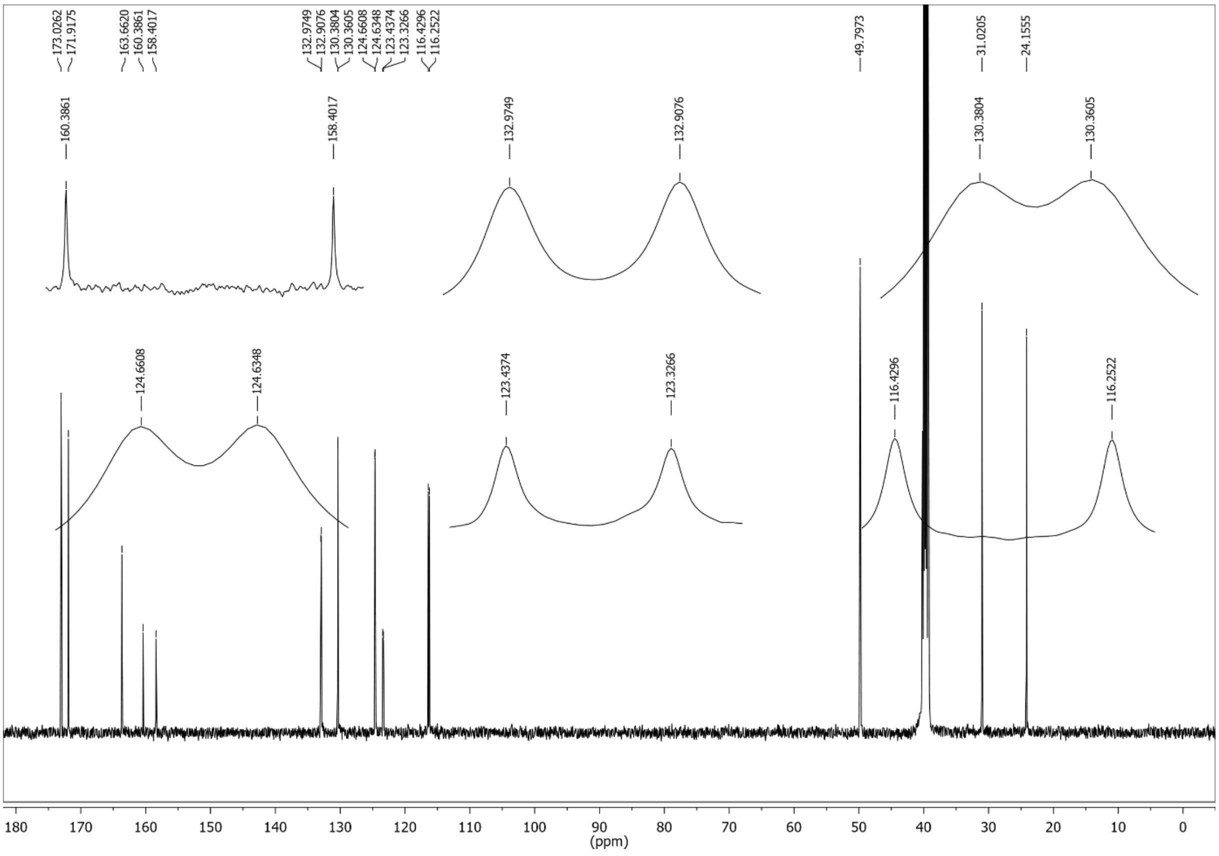

^{1}H and ^{13}C NMR spectra of compound **10**

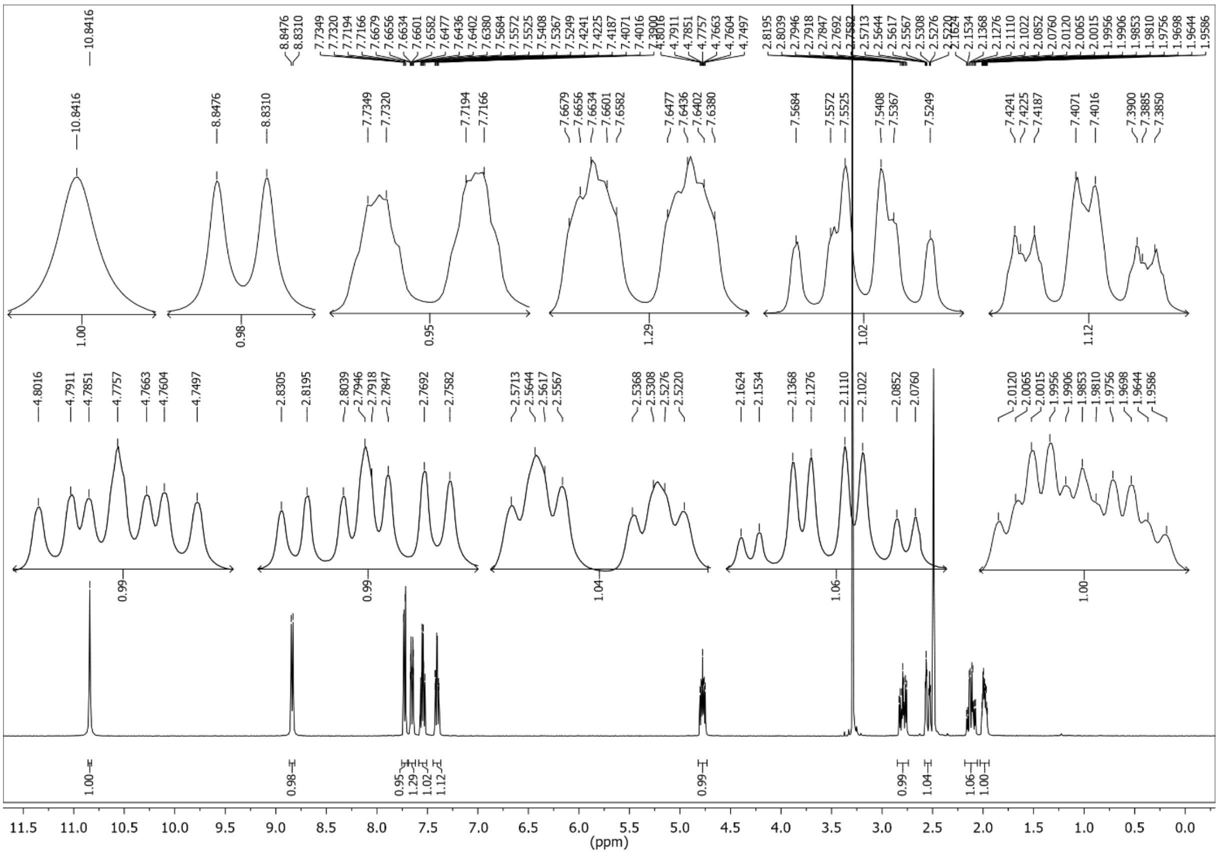

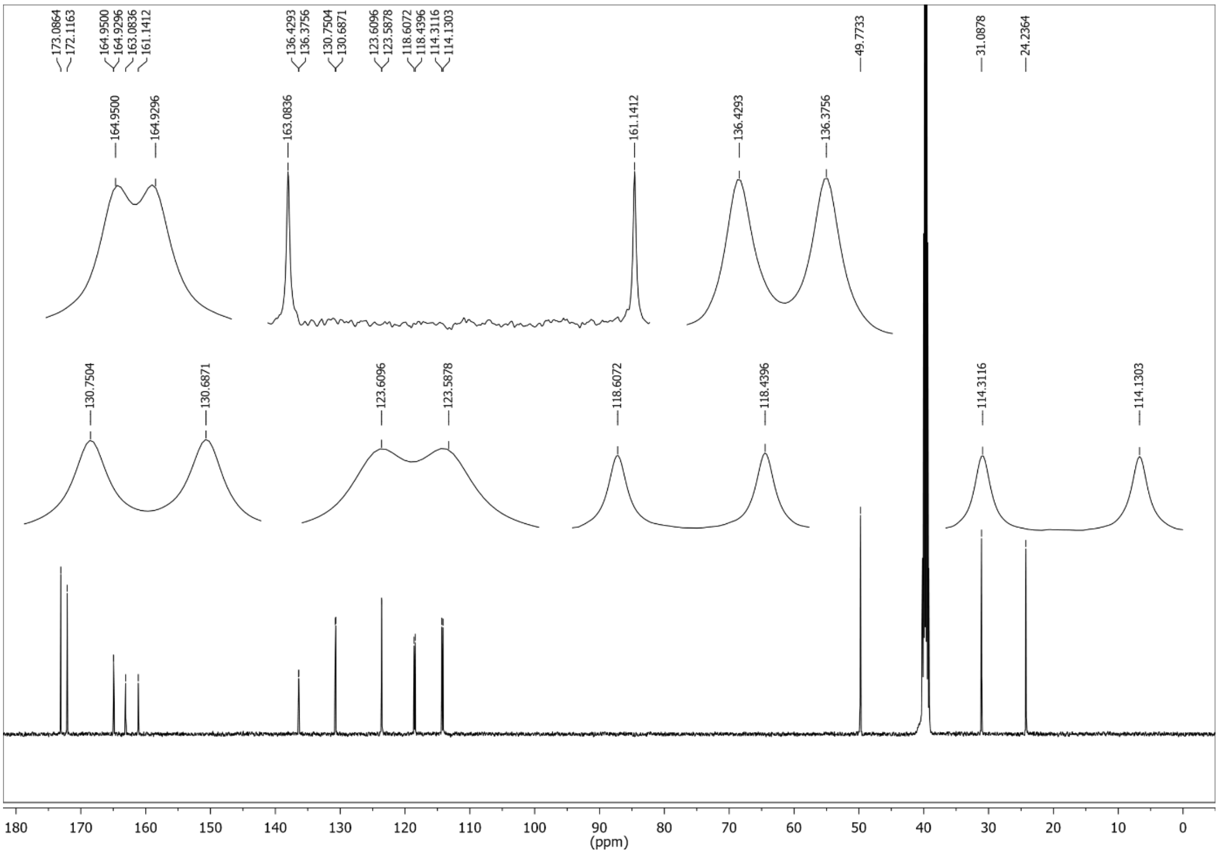

^{1}H and ^{13}C NMR spectra of compound **11**

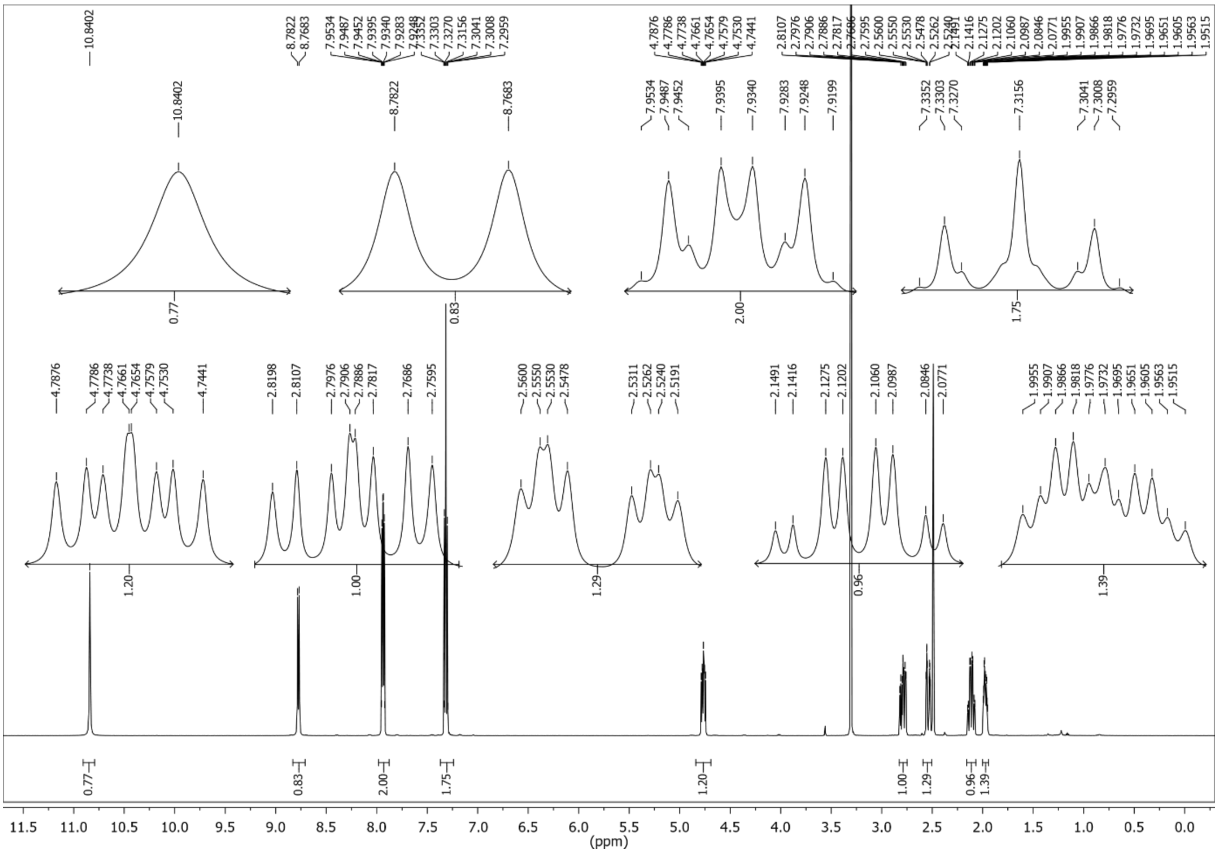

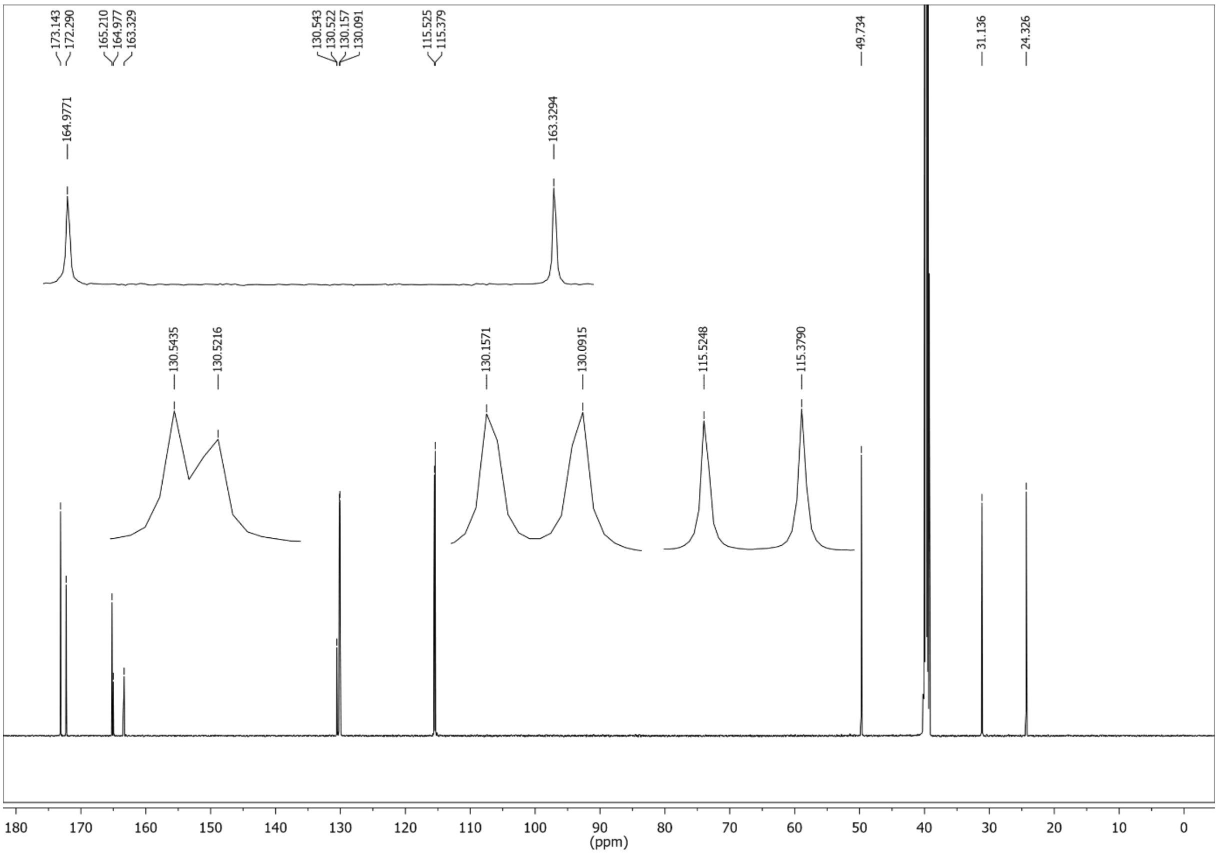

^{1}H and ^{13}C NMR spectra of compound **12**

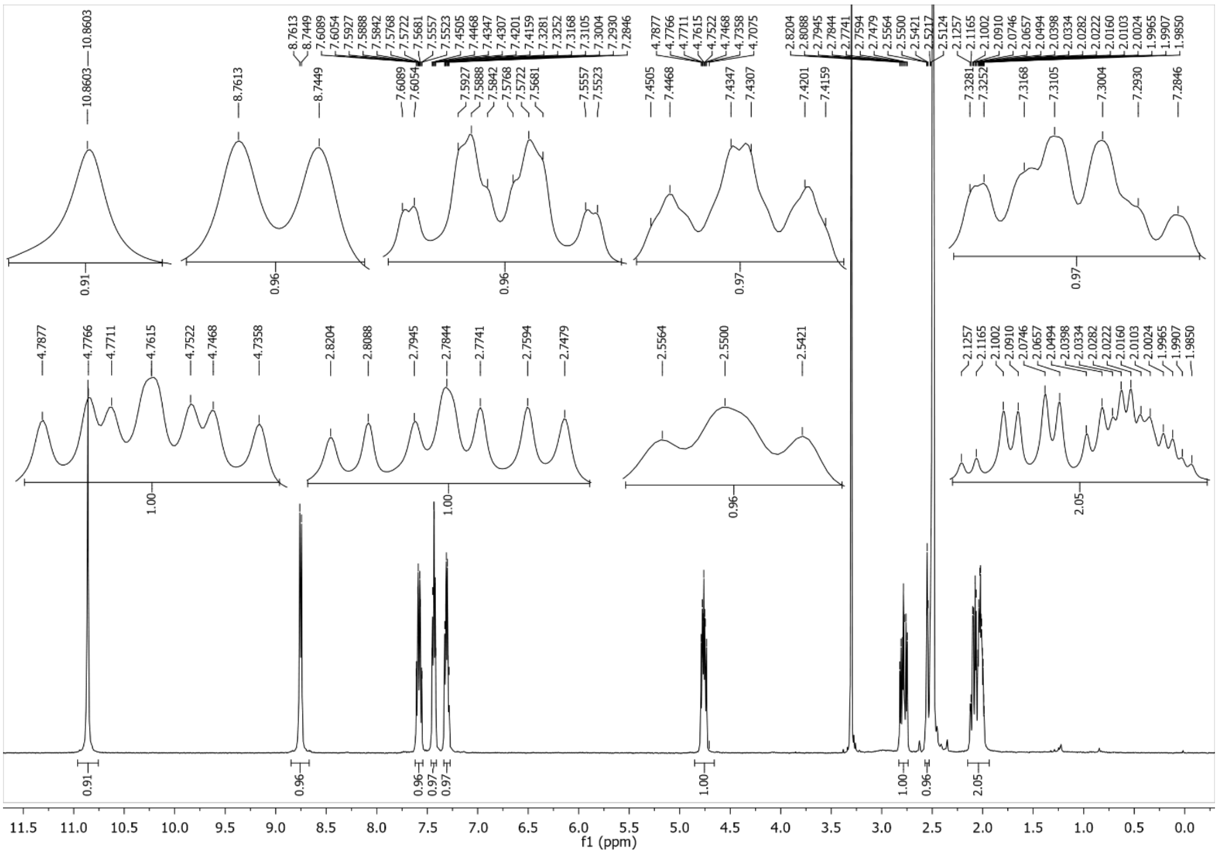

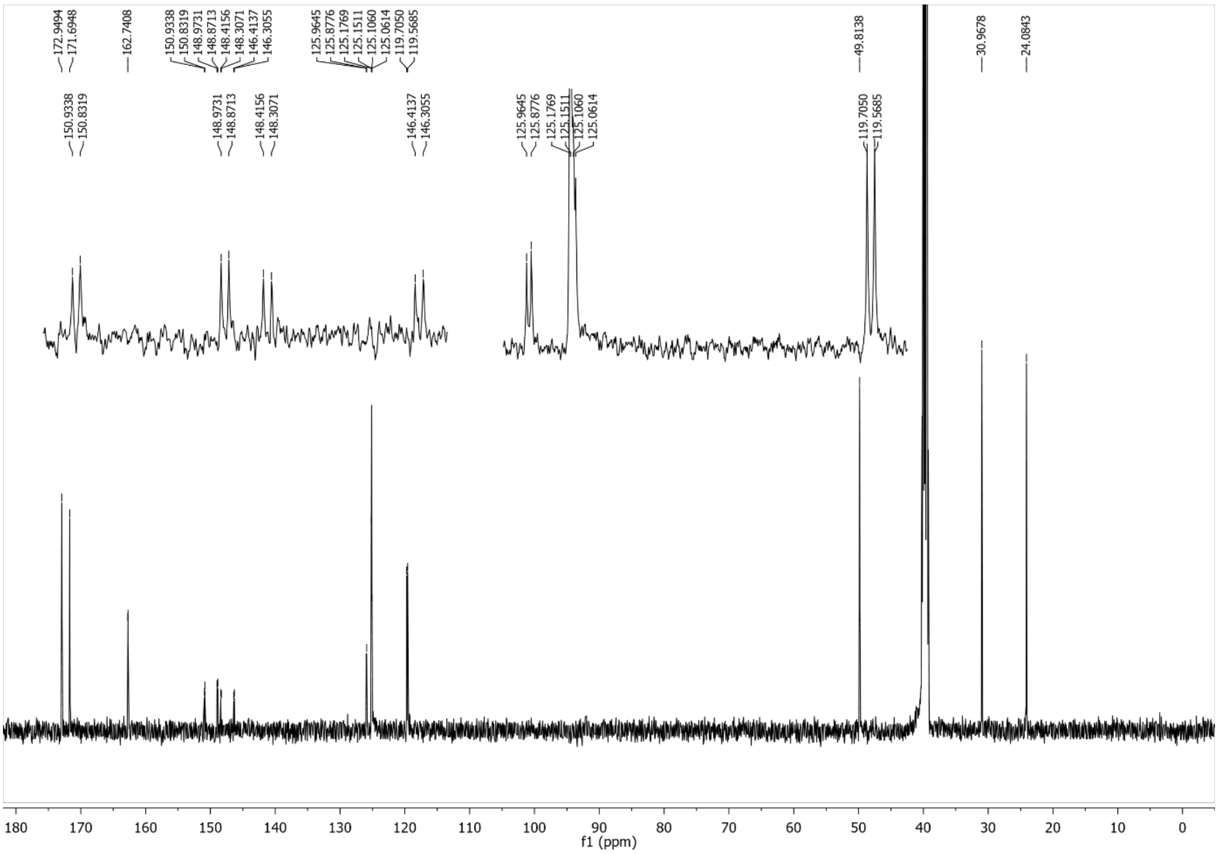

^{1}H and ^{13}C NMR spectra of compound **13**

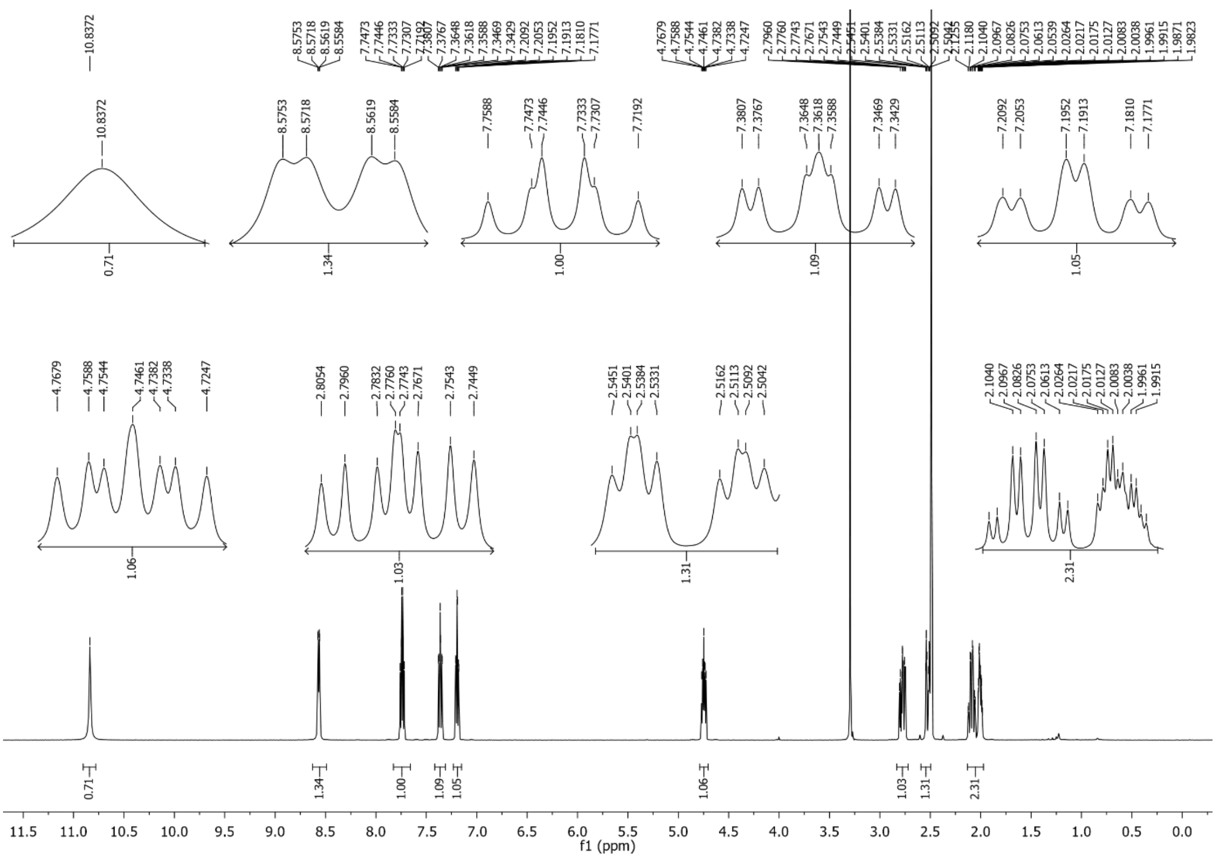

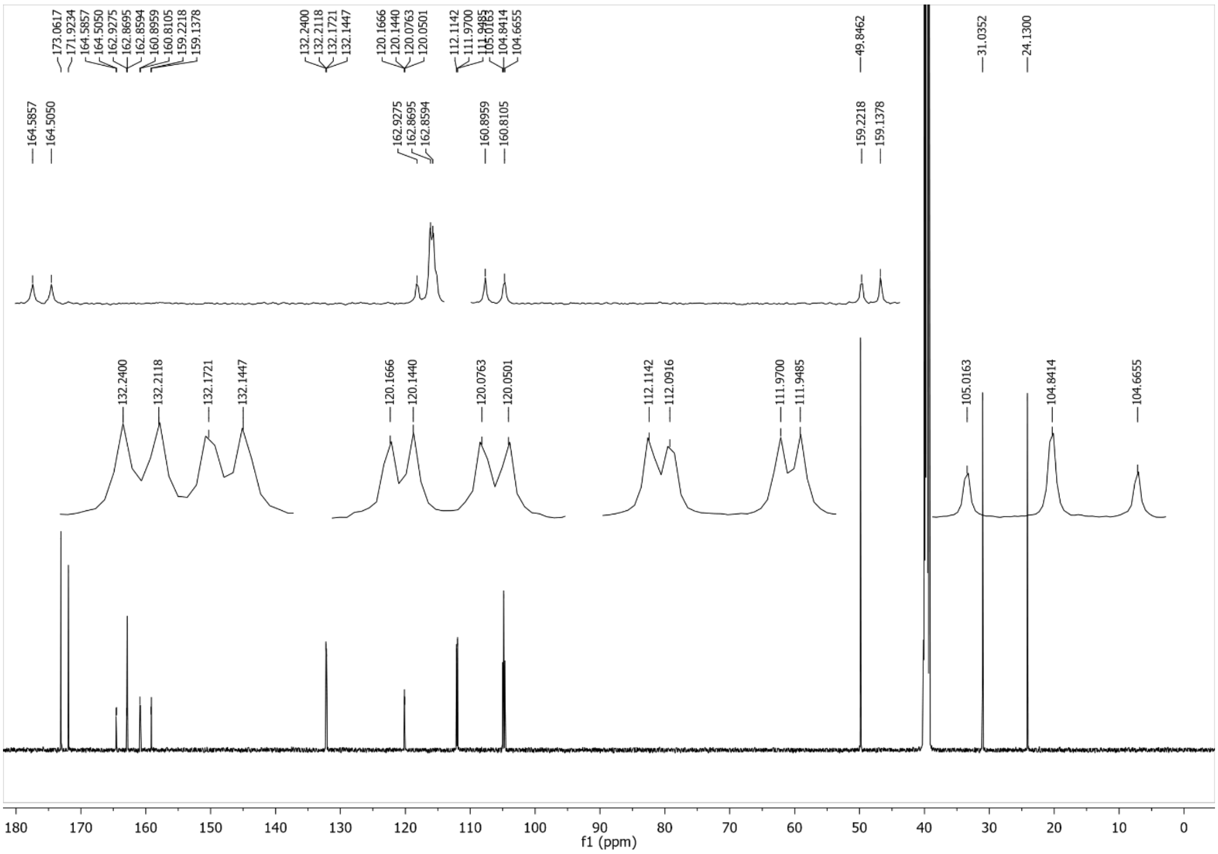

^{1}H and ^{13}C NMR spectra of compound **14**

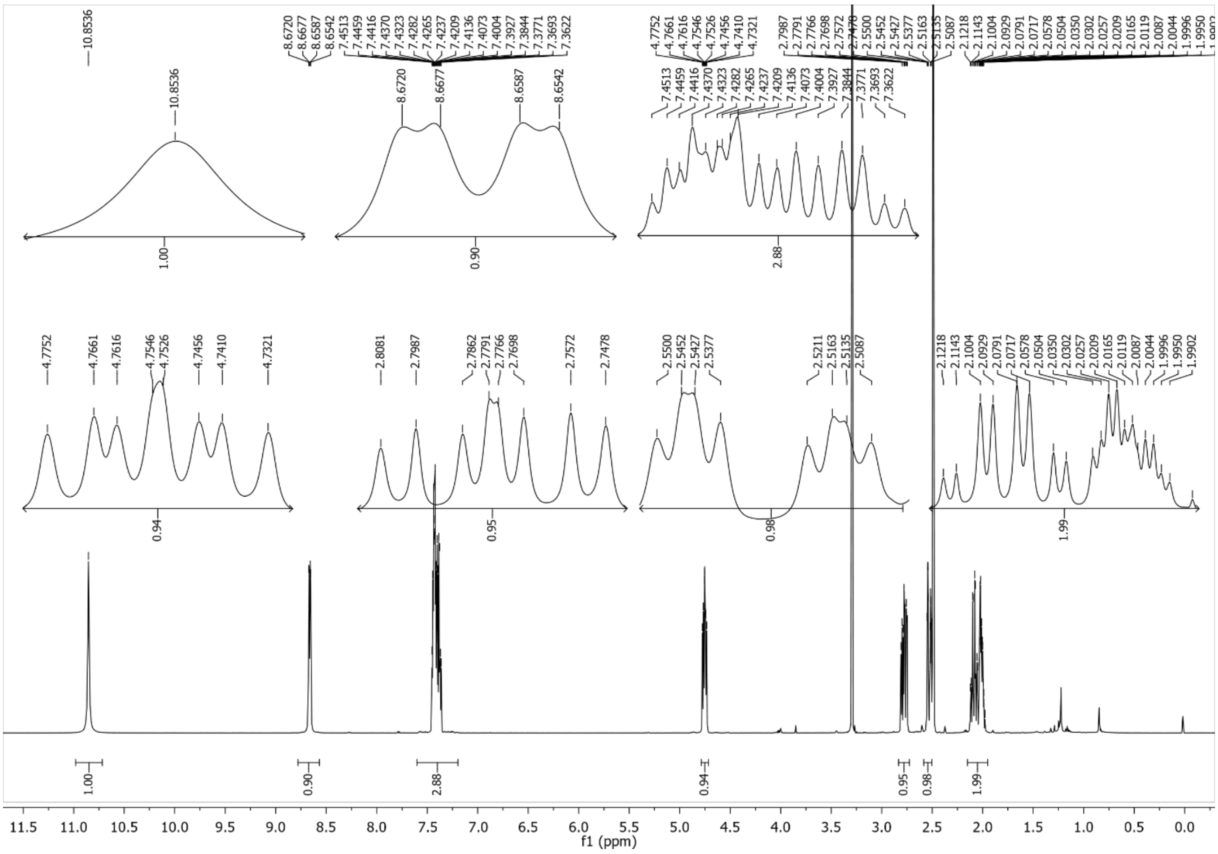

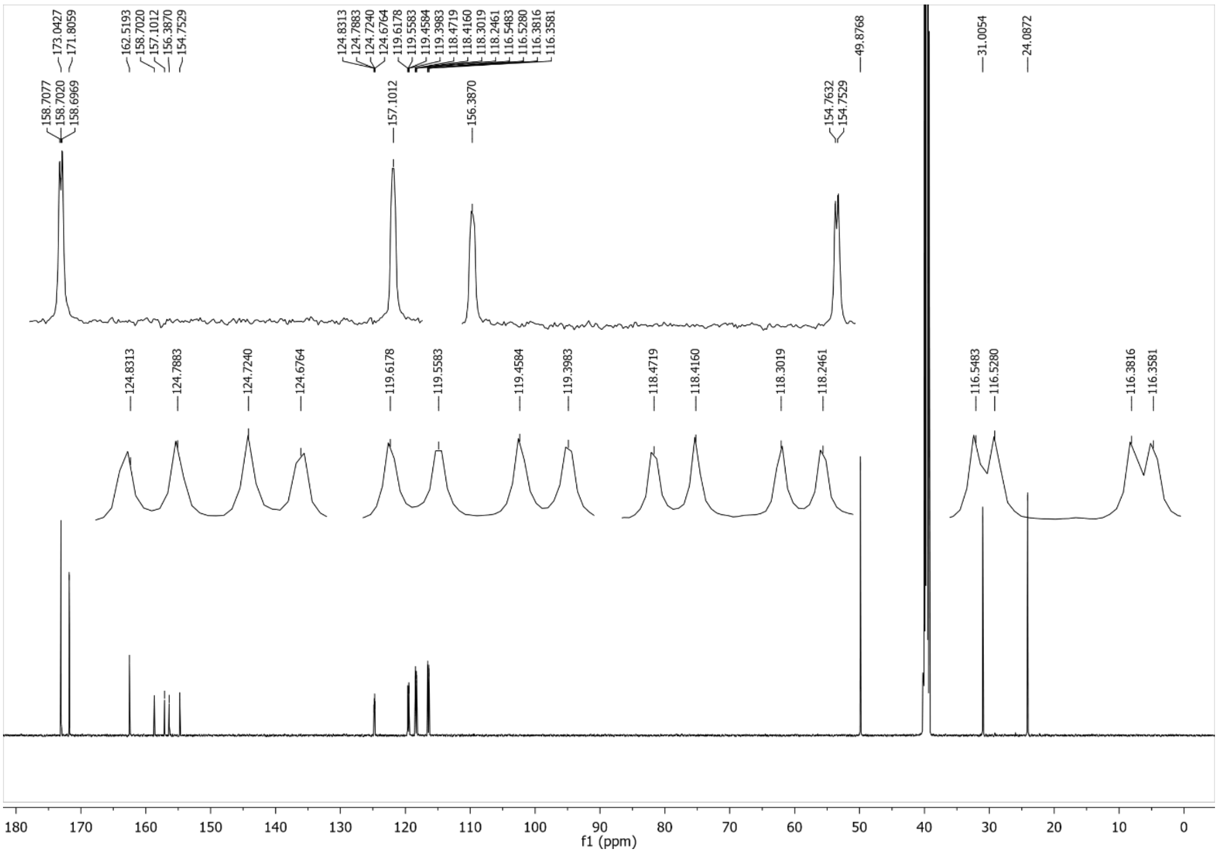

^{1}H and ^{13}C NMR spectra of compound **15**

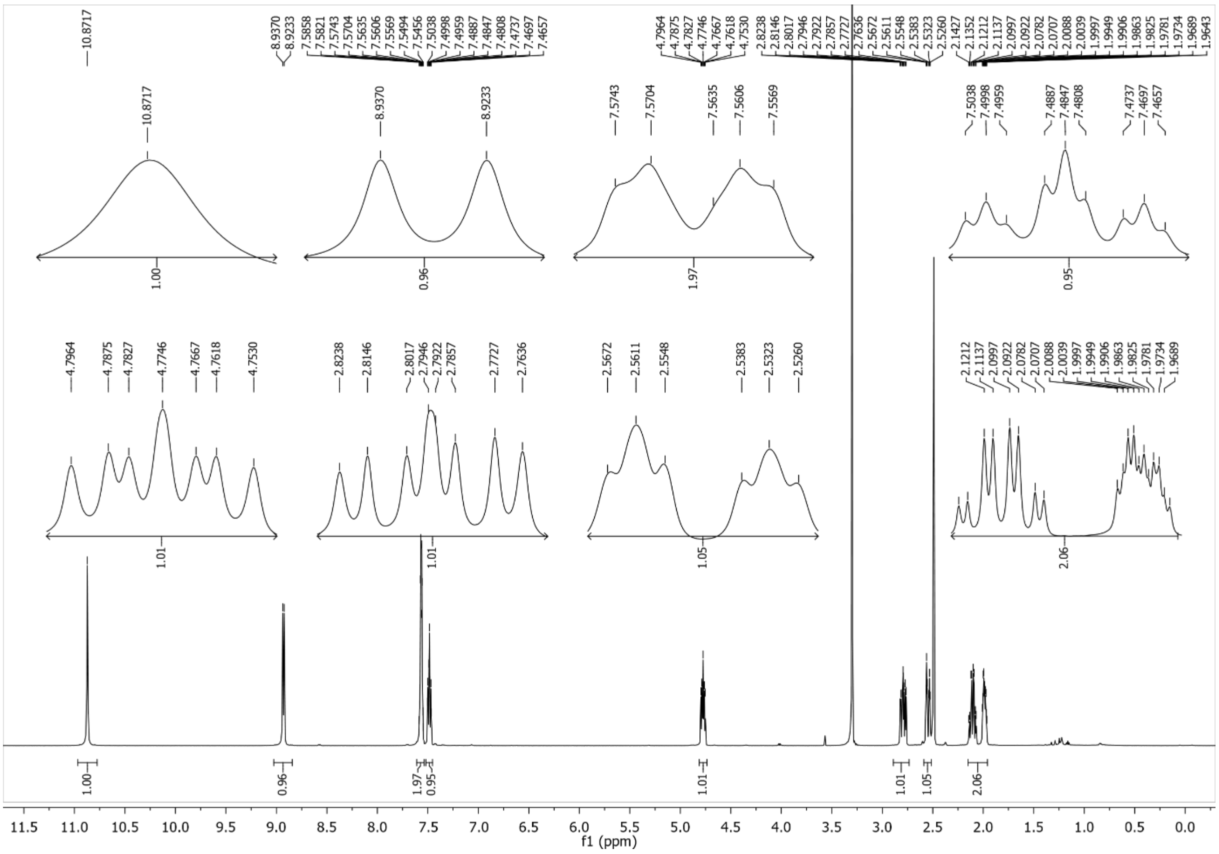

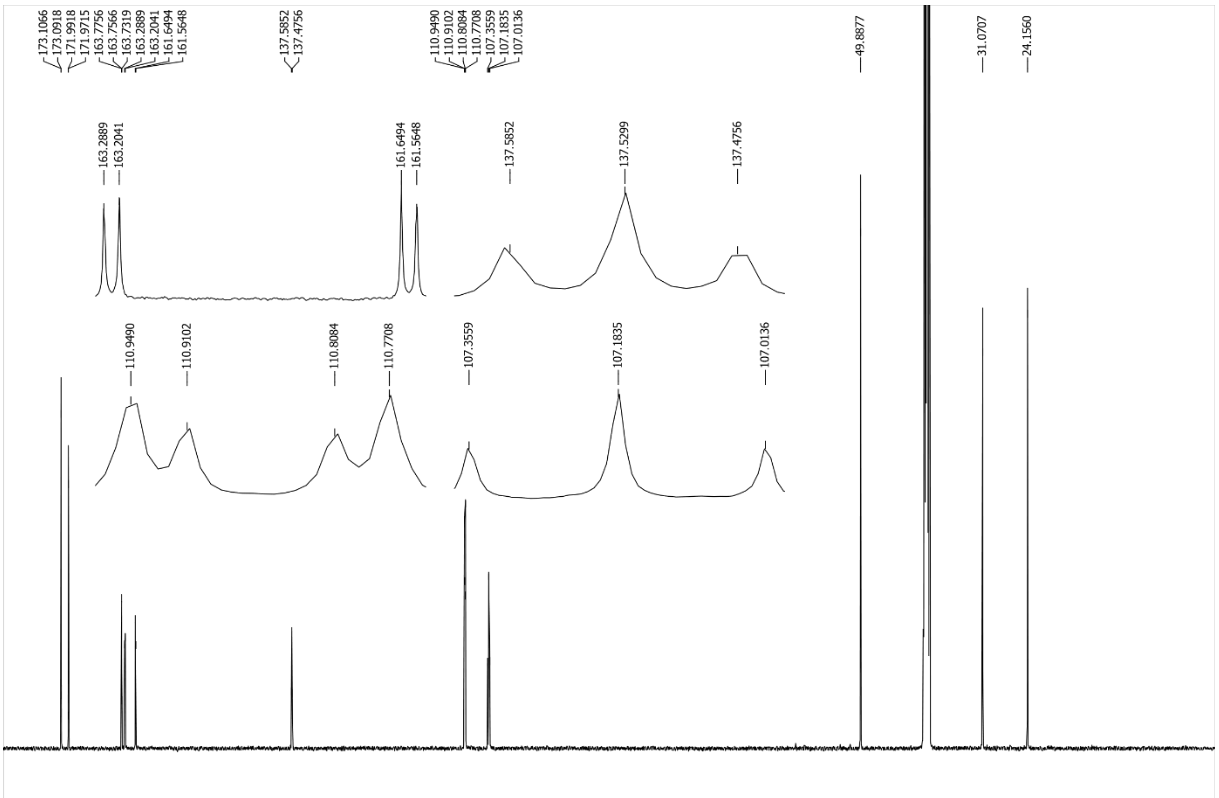

^{1}H and ^{13}C NMR spectra of compound **16**

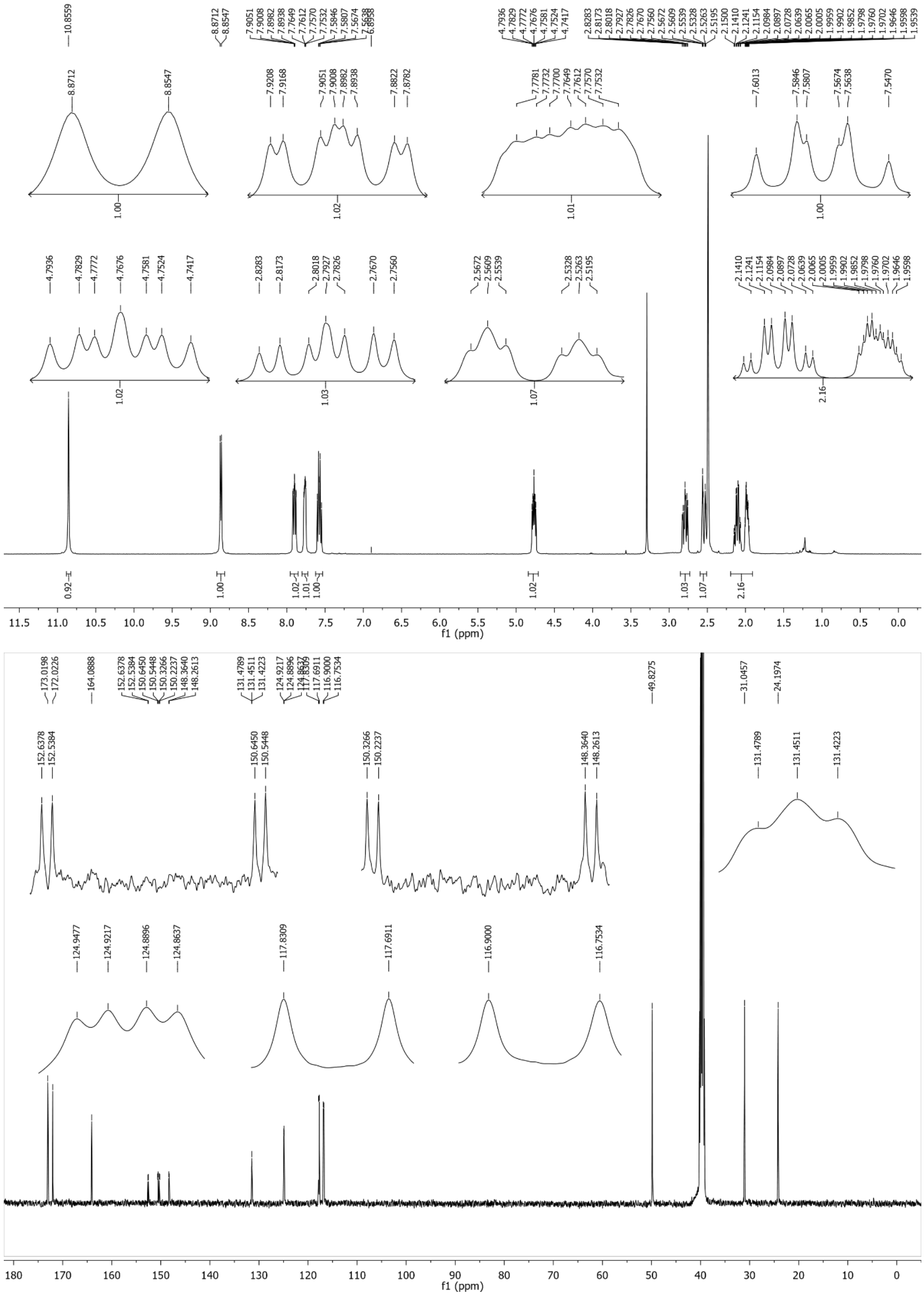

^{1}H and ^{13}C NMR spectra of compound **17**

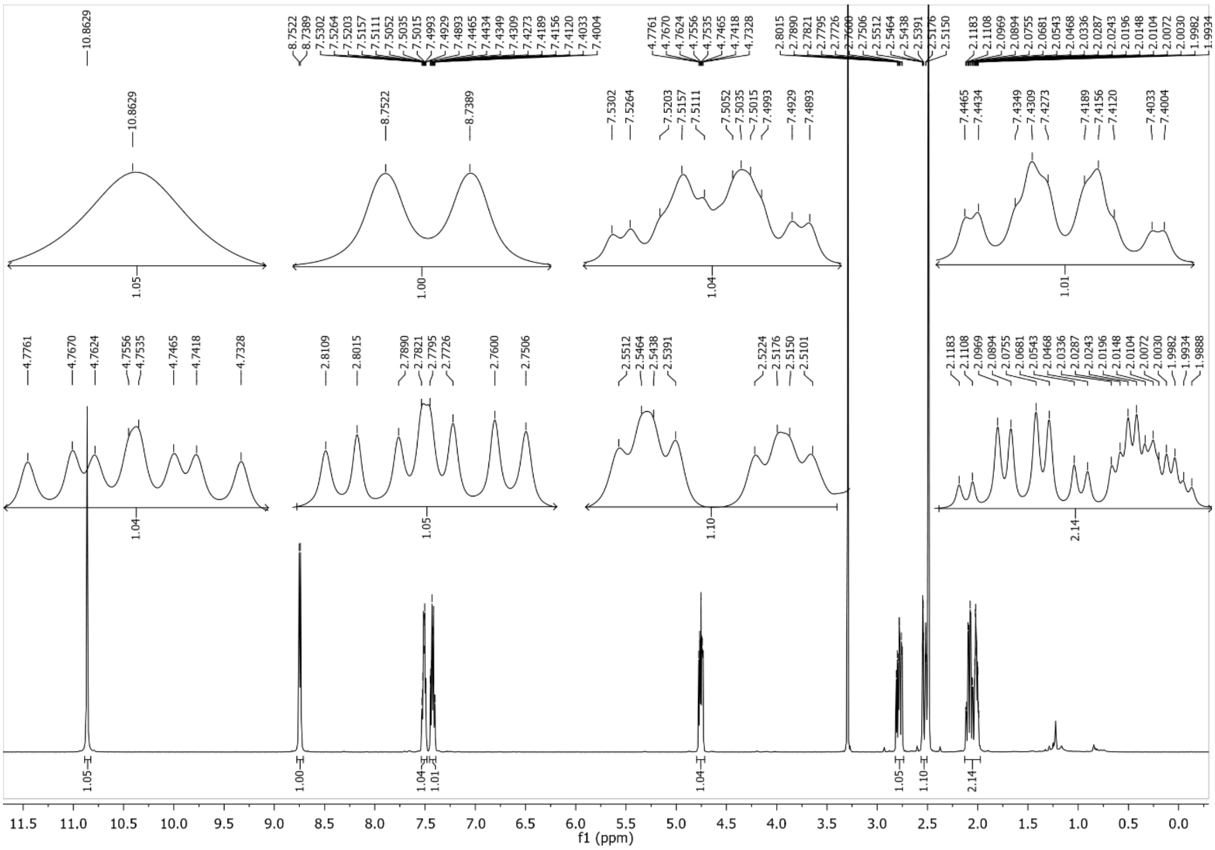

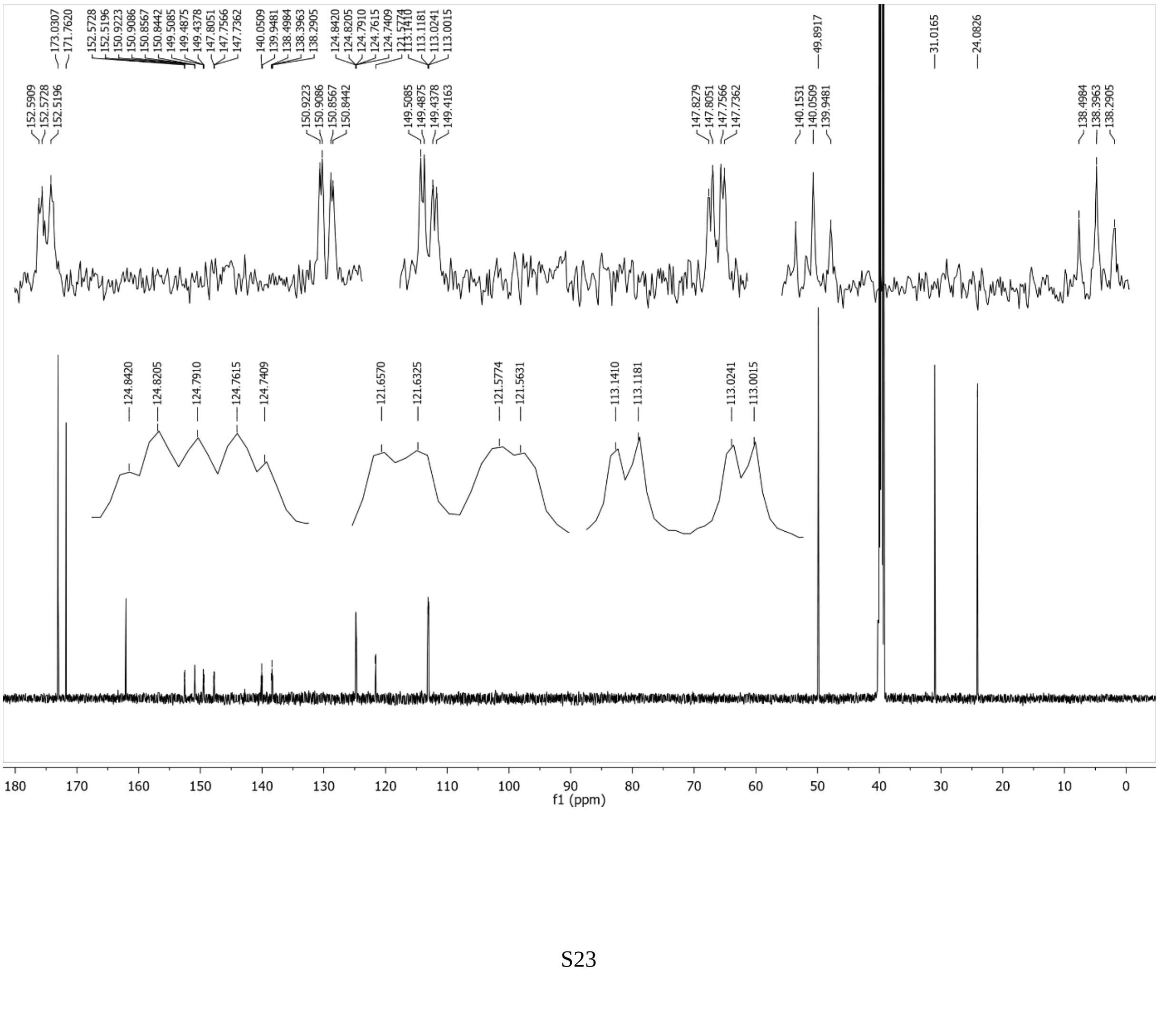

^{1}H and ^{13}C NMR spectra of compound **18**

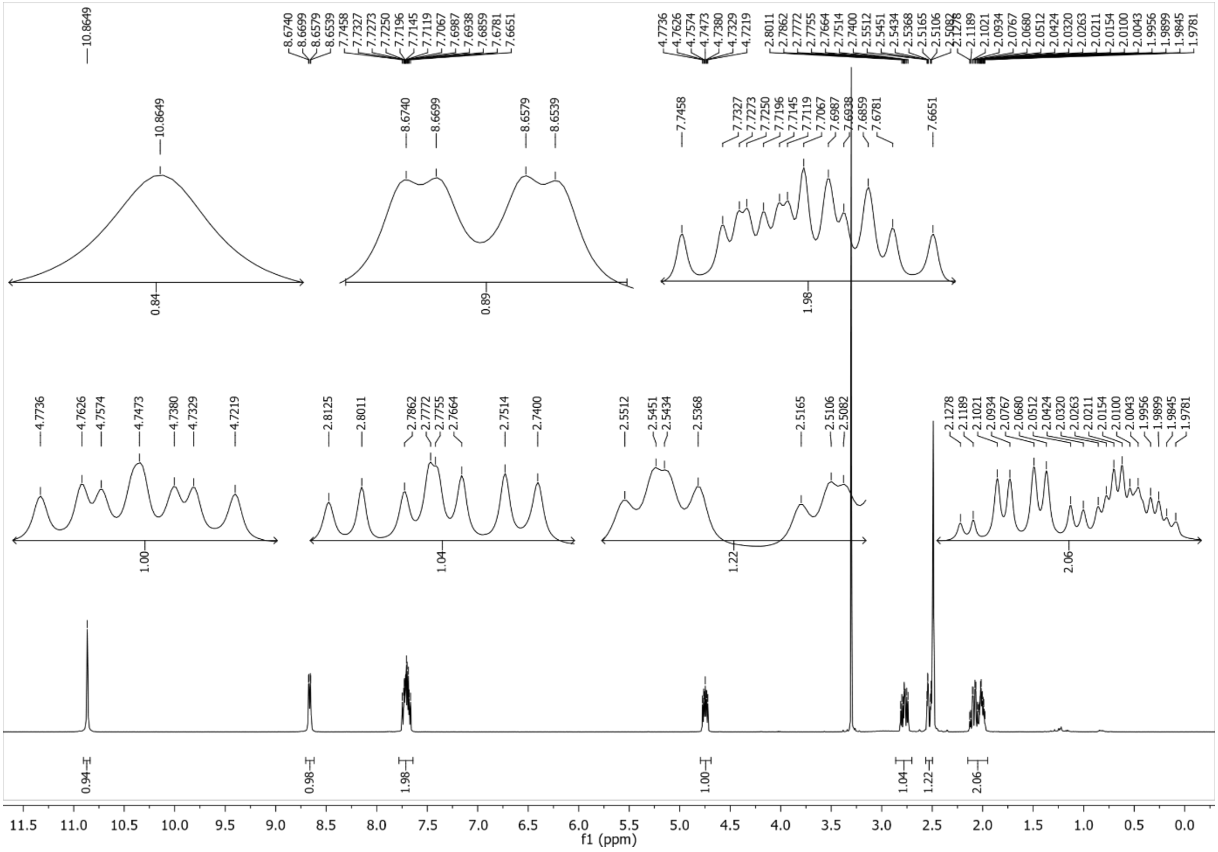

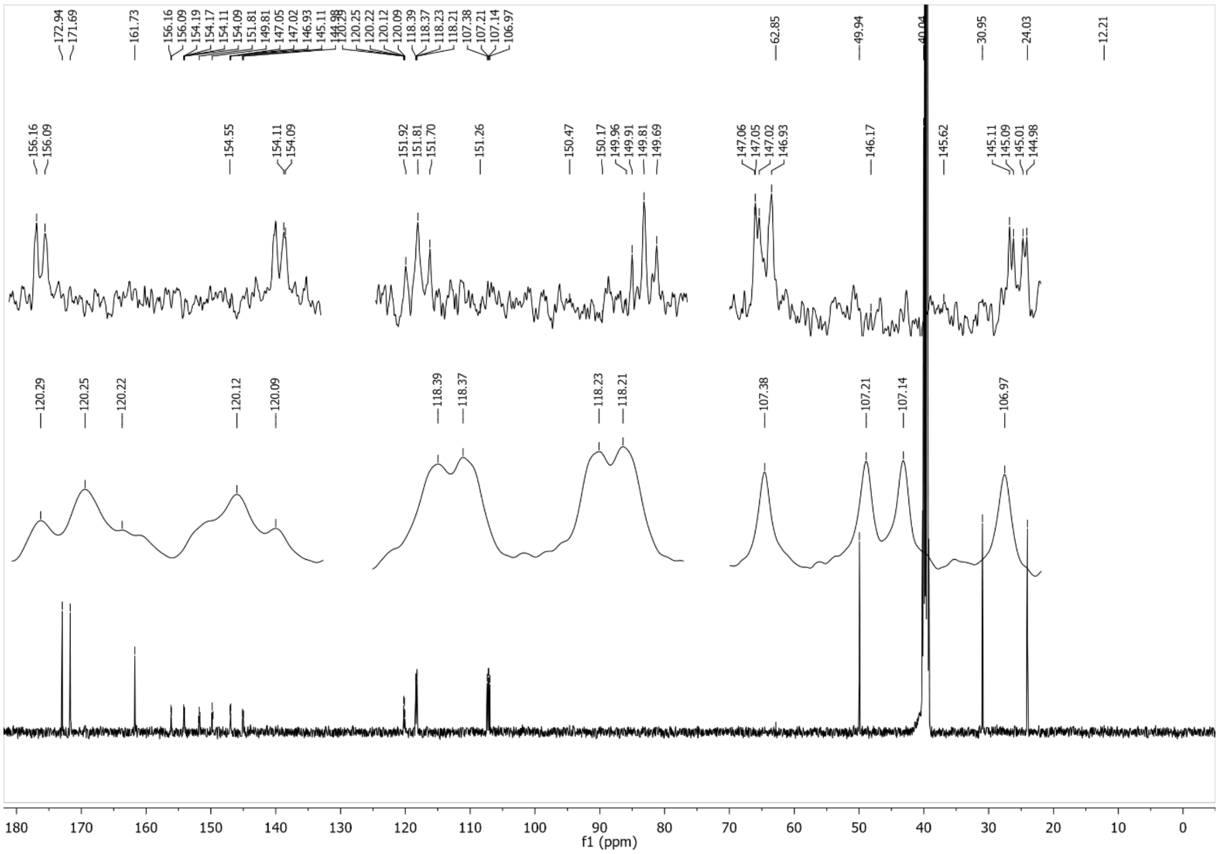

^{1}H and ^{13}C NMR spectra of compound **19**

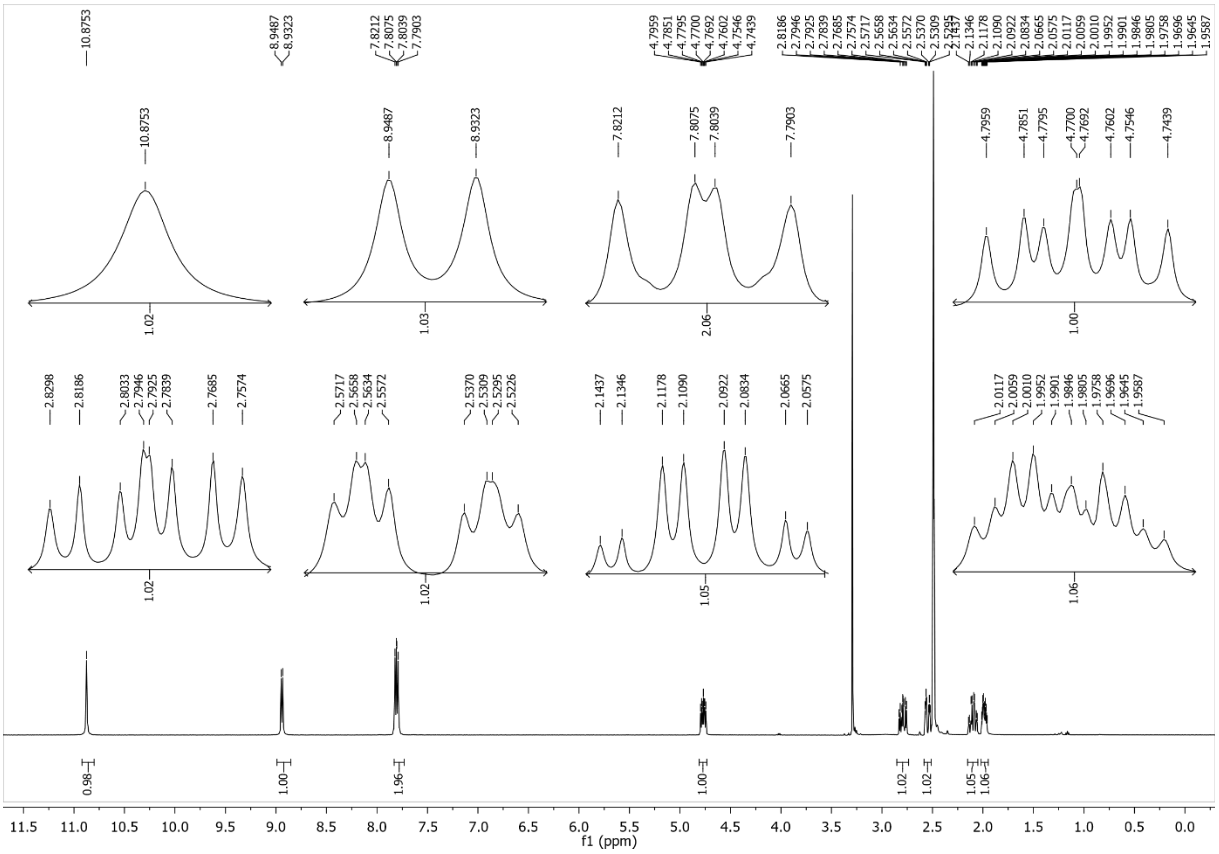

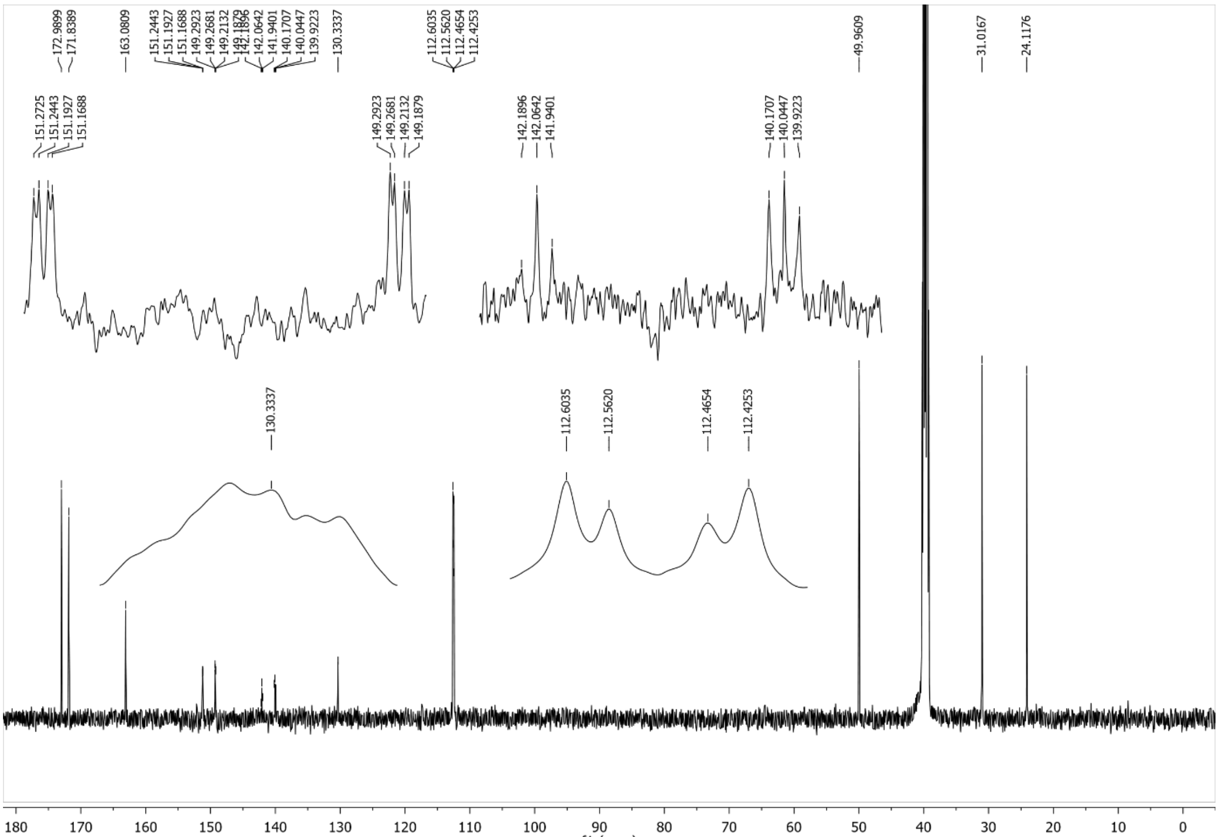

3. References

Heim C, Maiwald S, Steinebach C, Collins MK, Strope J, Chau CH, Figg WD, Gütschow M, Hartmann MD. On the correlation of cereblon binding, fluorination and antiangiogenic properties of immunomodulatory drugs. *Biochem Biophys Res Commun.* 2021;534:67–72.

Burslem GM, Ottis P, Jaime-Figueroa S, Morgan A, Cromm PM, Toure M, Crews CM. Efficient synthesis of immunomodulatory drug analogues enables exploration of structure-degradation relationships. *ChemMedChem.* 2018;13:1508–1512.

Ambrożak A, Steinebach C, Gardner ER, Beedie SL, Schnakenburg G, Figg WD, Gütschow M. Synthesis and antiangiogenic properties of tetrafluorophthalimido and tetrafluorobenzamido barbituric acids. *ChemMedChem*. 2016;11:2621−2629.

Steinebach C, Bricelj A, Murgai A, Sosič I, Bischof L, Ng YLD, Heim C, Maiwald S, Proj M, Voget R, Feller F, Košmrlj J, Sapozhnikova V, Schmidt A, Zuleeg MR, Lemnitzer P, Mertins P, Hansen FK, Gütschow M, Krönke J, Hartmann MD. Leveraging ligand affinity and properties: Discovery of novel benzamide-type cereblon binders for the design of PROTACs. *J Med Chem.* 2023;66:14513–14543.
